# Methods of porous and foamed structure formation in geopolymer-based materials: a comprehensive review

**DOI:** 10.1039/d6ra05119g

**Published:** 2026-09-28

**Authors:** Kristina Goryunova, Yunis Gahramanli

**Affiliations:** a Azerbaijan State Oil and Industry University Baku Azerbaijan kristina.qoryunova.i@asoiu.edu.az

## Abstract

Porous geopolymer-based materials are increasingly being considered as solutions for thermal insulation, lightweight construction, adsorption, catalysis and filtration. Existing reviews have mainly focused on individual fabrication methods, porous composites or specific areas of application, whilst direct comparisons of the main methods for creating porous structures, and how they influence pore architecture, remain limited. This review compares direct foaming, moulding using reactive emulsion templates, microwave-assisted foaming, the incorporation of lightweight fillers, additive manufacturing, replica processing and the use of sacrificial templates within a unified processing–structure–properties framework. Particular attention is paid to the interaction between pore formation, pore architecture, fresh-state rheology and geopolymerisation kinetics, which determines pore retention, morphology, connectivity and homogeneity. Analysis shows that direct foaming is the most practical and scalable method for producing low-density thermal insulation materials, whilst lightweight fillers provide an optimal balance between strength and density. Reactive emulsion templating, replica processing and the use of sacrificial templates are better suited to creating the interconnected pore networks required for adsorption, catalysis and filtration. Additive manufacturing offers the greatest control over architecture but remains limited by processing complexity, production speed and costs, whilst microwave-assisted foaming enables rapid processing but faces scaling challenges. The main contribution of this review lies in the development of a practice-oriented framework for selecting technological methods for forming porous structures depending on the desired pore architecture, as this is a determining parameter on which the material's performance characteristics and scope of application depend.

## Abstract 2

This review provides a structured overview of the main methods for creating porous structures in geopolymers.

## Graphical abstract

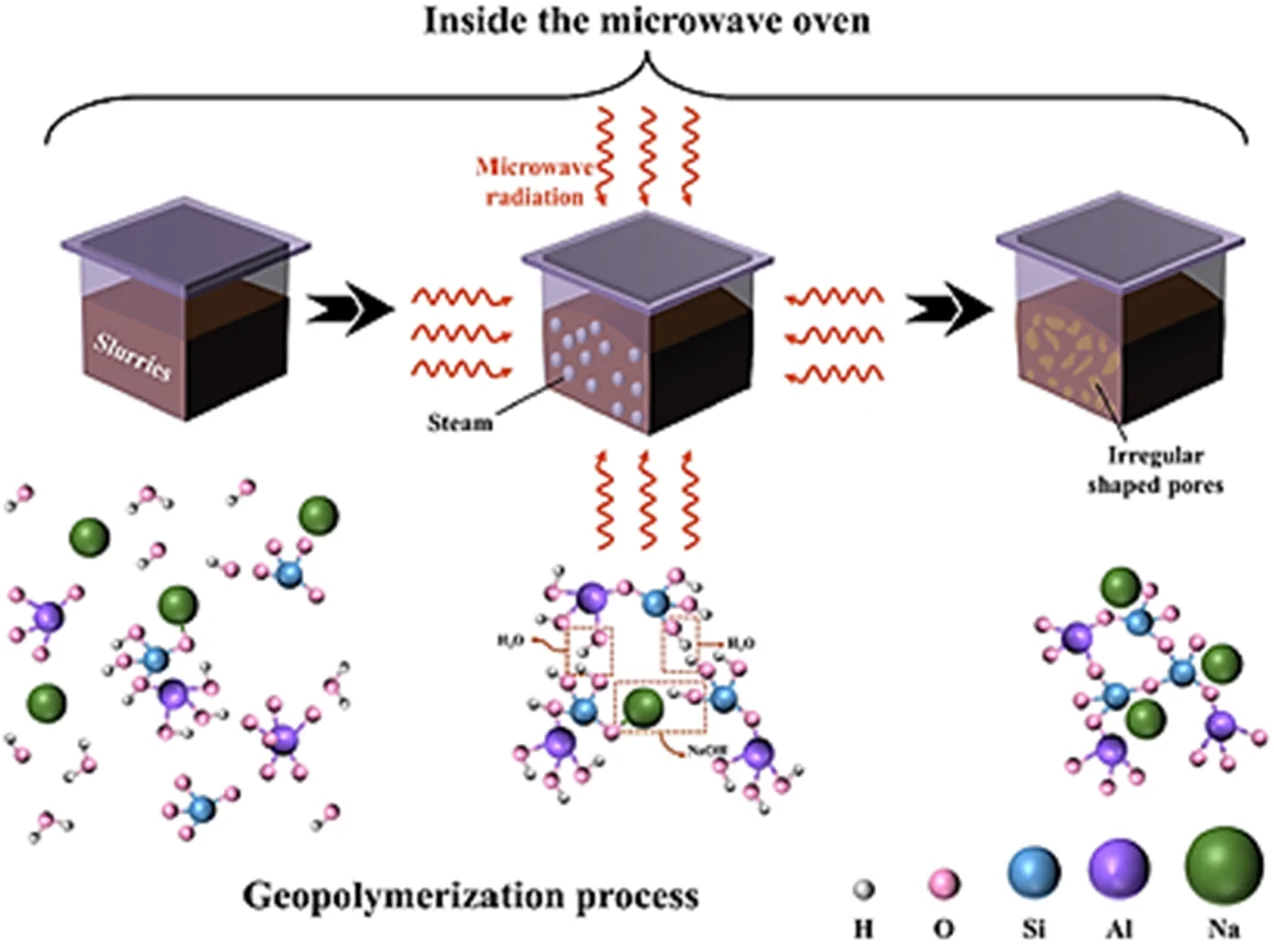

## 1. Introduction

There is growing pressure on the construction industry to decrease its environmental effects, while retaining the mechanical performance, durability and cost needed for large scale infrastructure. Concrete is still primarily a cement-based material, the most common binder used in concrete is ordinary Portland cement. It has large CO_2_ emissions and considerable energy usage during manufacture. Moreover, traditional concrete consumes a significant amount of natural aggregate, usually over 70% by volume.^1^ The global aggregate demand has been predicted to exceed 66 billion tonnes by 2025,^2^ causing more anxiety about resource depletion, land disturbance and ecological degradation. Concrete is one of the most utilised materials in building globally,^3^ thus measures to reduce its environmental impact have become a major research focus, such as the use of extra cementitious materials, industrial by-products, recycled or synthetic aggregates^4^ and alternative low carbon binders such as geopolymers.^5^

Concurrently, the need for multifunctional and energy-efficient construction materials is growing. In industrialised nations, buildings account for a considerable proportion of overall energy consumption and CO_2_ emissions.^6,7^ Many national initiatives are now aimed at reducing heat loss *via* building envelopes.^8^ Lightweight porous materials are therefore crucial for decreasing structural dead load as well as boosting thermal insulation, acoustic absorption and interior comfort.^9–11^ Foam concrete has already been widely used^12–17^ due to its low density, thermal insulation capability and functional benefits compared with conventional dense concrete. These features are due to the existence of air spaces or pores in the hardened matrix.^18^ However, the typical foam concrete mainly depends on Portland cement, which restricts the sustainability benefit. This led to the development of geopolymer-based foams and porous inorganic materials as an alternative solution with less embodied carbon and a wider range of raw ingredients.^14–18^

The synthesis of geopolymers is based on the activation of aluminosilicate precursors, such as metakaolin,^19–28^ fly ash, slag or calcium aluminosilicate materials,^28–35^ with alkaline or acidic activating solutions. During the geopolymerisation process, reactive silicate and aluminate species dissolve, re-organise and polycondense to produce a rigid inorganic network. This is commonly referred to as an amorphous alkali aluminosilicate gel which links unreacted particles and secondary phases.^36^ Geopolymer-based materials can achieve mechanical properties comparable or higher than those of ordinary Portland cement systems,^15,37,38^ depending on precursor chemistry and activator composition, allowing the incorporation of industrial residues and waste-derived raw materials.^15,39,40^ These properties make geopolymers interesting for the manufacture of low-carbon building materials and useful porous structures.

Beyond their potential environmental advantages, geopolymer-based binders offer several processing characteristics that make them attractive matrices for porous materials. Their fresh state rheology can be adjusted through precursor selection, activator composition, liquid-to-solid ratio, soluble silicate content and mineral additives. Their setting rate can also be modified over a relatively broad range, allowing pore-forming events to be coupled with matrix hardening. In addition, geopolymer slurries can accommodate gas-releasing agents, surfactants, lightweight fillers, emulsions, sacrificial particles and printable rheology modifiers. Unlike sintered ceramic foams and ordinary Portland cement, many geopolymer-based porous materials can be produced at low or moderate curing temperatures (80 °C), which reduces energy consumption and carbon dioxide emissions into the atmosphere, while retaining high thermal resistance and non-flammability compared with organic polymeric foams.^41–46^ These features provide a flexible platform for tailoring density, pore connectivity, thermal conductivity, mechanical strength and high specific surface area for different applications.

Foamed, aerated, cellular or porous geopolymer, *i.e.*, geopolymer foam concrete, is generated by creating gaps in a geopolymer paste, mortar or composite system.^41–45^ Depending on the kind of precursor, activator chemistry and pore design, geopolymer foams can provide enhanced strength at the same density and better thermal insulation performance than Portland cement-based foam concretes.^41–45^ But porosity is not the only factor that determines their qualities. Pore size, shape, connectivity and spatial distribution substantially affect the compressive strength, thermal conductivity, permeability, water absorption and durability. For example, closed and evenly dispersed holes are typically favourable for thermal insulation, whereas open and inter-connected pores are needed for filtration, adsorption, catalysis, and mass transfer. Thus, the main problem is not just to make a highly porous material, but to design the pore structure for a specific purpose.

Porous geopolymer materials have been reported to be fabricated using direct foaming, reactive emulsion templating, microwave-assisted foaming, embedding of lightweight or porous fillers, additive manufacturing, replica processing and sacrificial filler processes.^46^ Each approach produces porosity in a different way. The processes employed are direct foaming (gas generation or air incorporation within a setting slurry), microwave foaming (rapid water vapour expansion), reactive emulsion templating (removable liquid droplets), lightweight filler incorporation (pre-formed hollow or porous inclusions), replica and sacrificial filler methods (transfer of geometry from removable templates) and additive manufacturing (digitally designed pore architectures). Such distinctions are relevant because the manufacturing technique impacts not only the quantity of porosity, but also its stability, connectivity and effect on mechanical and functional performance.

Previous reviews have made substantial contributions to the understanding of porous geopolymer-based materials, but they address the field from different and largely complementary perspectives. Bai and Colombo^46^ classified the principal processing routes for highly porous geopolymers and compared their basic properties, whereas Novais *et al.*^47^ focused mainly on the emerging applications of geopolymer foams. Zhang *et al.*^48^ considered porous geopolymer composites containing reinforcing, lightweight or functional secondary phases. More specialised reviews have examined directly foamed geopolymers for construction and insulation applications and extrusion-based 3D printing of geopolymers, with detailed emphasis on foaming agents, stabilisation, printability and rheology.^49,50^ However, these studies do not provide a unified cross-method assessment linking foaming methods, geopolymerisation kinetics, fresh-state rheology, resulting pore architecture, mechanical performance and scalability. Furthermore, these reviews do not provide researchers with clear guidance on which foaming method to choose in order to achieve a specific pore architecture.

The present review addresses this gap by evaluating direct foaming, reactive emulsion templating, microwave-assisted foaming, lightweight filler incorporation, additive manufacturing, replica processing and sacrificial templating within a common processing–structure–performance framework. The scope of this review is limited to fabrication routes in which pore architecture is deliberatively introduced and controlled in geopolymer-based matrices. The main innovation lies in the fact that the production of porous geopolymers is viewed as a problem of method selection, aimed at achieving a specific pore architecture which determines the material's subsequent application. Rather than comparing the methods only through total porosity or density, the review considers how pore-generation and matrix hardening mechanisms determine pore morphology, connectivity, accessibility and structural reliability. The resulting analysis identifies the trade-offs among architectural control, processing simplicity, mechanical performance and scalability and provides a practical basis for selecting a fabrication route for thermal insulation, lightweight construction, adsorption, catalysis, filtration and architected multifunctional components.

In this review the manufacturing of porous geopolymers is considered as an application-driven problem of method selection rather than a mere list of foaming processes. The framework is meant to aid in determining where each approach is most applicable, what its limits are, and what advancements are needed for porous geopolymer materials to transition from laboratory investigations to practical construction and functional applications.

## 2. Fundamentals of geopolymer formation and chemistry

The structure of geopolymers comprises a three-dimensional, irregularly ordered aluminosilicate network in which [SiO_4_]^−^ and [AlO_4_]^−^ tetrahedra are interconnected through shared oxygen atoms to form a sialate framework. When analysed by X-ray Diffraction (XRD), geopolymers exhibit broad amorphous halos similar to those observed in zeolitic materials, thus representing their non-crystalline analogues.^51^ Barbosa *et al.*^52^ described the geopolymer structure as a randomly arranged three-dimensional network of silicate and aluminate tetrahedra linked through common oxygen atoms. Owing to this structural resemblance, particularly regarding ion-exchange capacity and metal-ion accommodation, geopolymers have been proposed as amorphous analogues of zeolites with potential for similar functional applications.^53^

Geopolymerisation is generally described as the reaction between an aluminosilicate or calcium aluminosilicate precursor and an alkaline or acidic activating solution.^54–56^ This process is often discussed mainly in relation to gel formation and final strength. However, for porous and foamed geopolymers, geopolymerisation also controls the fresh-state evolution of viscosity, yield stress and setting. These parameters determine whether bubbles, vapour pockets, liquid droplets or removable templates can be retained until the matrix becomes sufficiently rigid. Therefore, the chemistry of geopolymer formation is directly connected to pore formation and foam stability.

The essential stages of geopolymerisation can be summarised as follows:

(1) Dissolution/hydrolysis. The aluminosilicate precursor is attacked by the alkaline solution in a strongly basic medium. Monovalent cations (Na^+^, K^+^) from the activator replace surface-bound H^+^ ions, promoting the cleavage of Si–O–Si and Si–O–Al bonds and yielding soluble silicate and aluminate species, primarily Si(OH)_4_ and Al(OH)_4_^−^.

(2) Reorganisation. The dissolved species subsequently undergo condensation reactions, forming aluminosilicate oligomers. This stage plays a decisive role in shaping the microstructure and pore distribution of the final material.

(3) Polycondensation. When the concentration of aluminate species becomes sufficient to destabilise the silicate solution, gel formation begins. As condensation progresses, the gel phase evolves into a rigid, cross-linked aluminosilicate network, resulting in the hardening of the material.

Thus, porous geopolymer formation should be regarded as a coupled chemical and pore-forming process. The precursor and activator determine dissolution rate, gel formation and setting, while the foaming or templating route determines how pores are generated. The final pore architecture is formed only when these two processes are synchronised.

### 2.1. Synthesis of geopolymers

#### 2.1.1. Sources of aluminosilicates

Clays, especially kaolinite-rich materials, are among the most studied aluminosilicate sources for geopolymer synthesis. Thermal treatment of kaolinite produces metakaolin, a reactive amorphous aluminosilicate phase suitable for alkaline activation.^57^ Metakaolin is useful because of its relatively controlled composition and high reactivity, but its lamellar particle morphology can increase water demand and paste viscosity. This becomes important in foamed systems because water demand and viscosity directly affect bubble dispersion and pore stability.

Industrial by-products such as fly ash, blast furnace slag, silica fume and red mud have also been widely investigated as alternative precursors.^57,58^ Their use supports waste valorisation and the development of lower-carbon binders, but their composition and reactivity vary strongly. Fly ash is dominated by SiO_2_ and Al_2_O_3_ with variable CaO, Fe_2_O_3_ and MgO contents depending on coal source and combustion conditions. Class F fly ash is generally low in calcium and may react more slowly, whereas Class C fly ash contains more calcium and can show self-cementitious behaviour.^59^ In foamed geopolymers, these differences influence not only final gel chemistry, but also setting rate, viscosity evolution and foam stability.

Blast furnace slag is a calcium-rich glassy precursor produced during ironmaking.^60^ When granulated, it can be efficiently activated and is widely used to improve reactivity and mechanical strength.^61^ In porous systems, slag can be beneficial because calcium-rich reaction products may increase early stiffness and help retain bubbles. However, excessive early stiffening can also shorten the processing window and restrict homogenous foaming. Therefore, slag content should be balanced according to both strength requirements and foam stability.

Red mud, or bauxite residue, is generated during alumina production and contains Fe_2_O_3_, Al_2_O_3_, SiO_2_, CaO, Na_2_O and TiO_2_, together with residual alkalinity.^62^ Its high pH, fine particle size and oxide composition make it a potential geopolymer precursor, but its variability and high iron content may complicate reaction control. Other residues, including silica fume, municipal solid waste incineration ash, waste glass and mining by-products, have also been explored.^63,64^ These materials often require blending with more reactive precursors such as metakaolin, fly ash or slag to achieve sufficient dissolution and network formation.

Biomass-derived ashes, including fly ash, rice husk ash and gasification-derived ashes, have recently attracted attention as a sustainable raw material for geopolymers.^65–67^ They can provide reactive silica, calcium or aluminosilicate phases, but their composition may be highly variable. Unburnt carbon, soluble salts and crystalline phases can modify water demand, setting behaviour and rheology. For porous geopolymers, this variability is especially important because small changes in viscosity or setting time can strongly alter pore size distribution and foam stability.

Natural volcanic materials such as volcanic ash, natural pozzolans and pumice are also suitable precursors where locally available.^68^ Their reactivity depends on glassy phase content fineness and chemical composition. Overall, many natural, industrial and biomass-derived aluminosilicate sources can be used for geopolymer synthesis. However, for porous geopolymer materials, precursor selection should not be based only on oxide composition or sustainability. Reactivity, particle morphology, calcium content, water demand and influence on rheology must also be considered because these factors control whether the developing matrix can preserve the desired pore structure.

#### 2.1.2. Activating solutions

Geopolymers are classified according to the chemistry of the precursor and the activating solution. There are two major categories of geopolymer systems, *i.e.*, alkali activated and phosphate, or acid activated. The activator influences pH, dissolving rate, availability of soluble silicate, gel type and setting behaviour. Thus, it regulates not just the ultimate matrix shape but also the fresh-state rheology that controls pore stability during foaming.

Alkali activation is the reaction of a silicate or aluminosilicate precursor with a very alkaline solution. The process involves dissolution, coagulation, polycondensation and production of hardened mineral phases, generally below 100 °C.^69–71^ Common activators are sodium or potassium hydroxides, silicates, carbonates, sulphates and aluminates.^70^ As alkali activators have a substantial expense and environmental impact, partial substitution of commercial alkali silicates using ashes, red mud, Bayer process liquid or other alternative sources has been examined.^72^ Activators can be used as an aqueous solution or as a solid component in one-part geopolymers formulations.^73^

Low-calcium alkali-activated systems are often dominated by sodium aluminosilicate hydrate, N–A–S–H, gel. Their typical silicon surroundings are SiQ^4^(2Al) and SiQ^4^(3Al), as illustrated in Fig. 1.^74^ In the SiQ^*n*^(*m*Al) nomenclature *n* is the number of bridging oxygens, while *m* is the number of surrounding [AlO_4_]^5−^ tetrahedra around silicon. These low-calcium geopolymers are very strongly cross-linked and are often classified as amorphous or pseudo-zeolitic systems.^70,74^

**Fig. 1:**
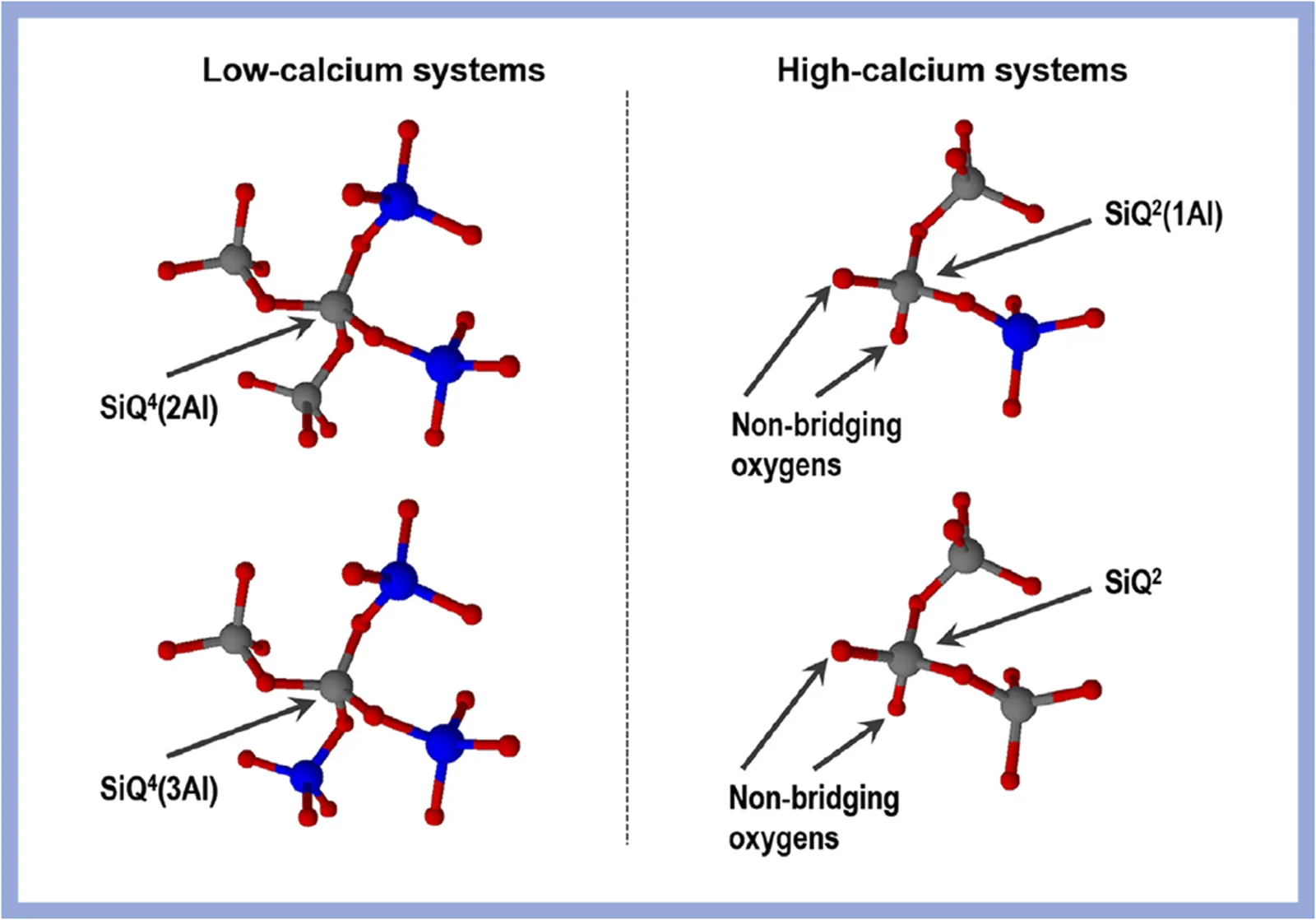
The predominant silicon environments in low-calcium alkali-activated materials (geopolymers) and high-calcium alkali-activated materials. The grey atoms in the figure represent silicon, red atoms oxygen, and blue atoms aluminium. This figure has been adapted/reproduced from ref. 74 with permission from Elsevier under the license number 6240770996663, copyright 2026.

In contrast, calcium-rich systems, in particular slag-rich binders, produce calcium–(sodium)–aluminosilicate–hydrate, C–(N)–A–S–H, gel. This gel comprises chain silicate units such as SiQ^2^ and SiQ^2^(1Al).^74,75^ Low-calcium geopolymers are predominantly stabilised by Si–O and Al–O bonding, whereas high-calcium systems have calcium-rich chain-like structures.^76,77^ This distinction is essential for porous materials since calcium-rich solutions can stiffen up early more quickly which may increase bubble retention but can also shorten the available time for mixing, expansion or casting.

The activator also has a direct influence on the foaming behaviour. High alkalinity can accelerate precursor dissolution and may accelerate gas-producing processes, while soluble silicate might increase viscosity and encourage faster gel formation. Such effects can be beneficial for foam stability if they assist preserve bubbles but harmful if the slurry becomes too stiff to harm expansion. Combinations of activators were shown to have a considerable influence on the development of yield-stress and stability of ultra-lightweight geopolymer foam concrete.^78^ The mixes with sodium silicate exhibited a quicker development of the yield stress and the combination of sodium silicate and Ca(OH)_2_ resulted in the most stable fresh foam, due to early rheological resistance which minimised settlement and bubble collapse.^78^

Acid-activated aluminosilicate binders have been reported in the literature, in particular phosphate-based geopolymers.^79–81^ Their reaction process is different from a common alkali activation. Early models suggested a connection between P–O tetrahedra of phosphoric acid and Al–O tetrahedral sites of metakaolin.^82–84^ Later, Davidovits proposed transitory Si–O–P–O–Al connections and subsequent polymerisation into AlPO_4_^−^ and SiO_2_-rich phases.^85^ Louati *et al.* reported a process of dealumination of metakaolin followed by reaction with PO_4_^3−^ tetrahedra and polycondensation to form a cohesive matrix.^86^ Mathivet *et al.* presented a similar sequence of dissolution, polycondensation and reaction completion.^87^ Kinetic investigations are still restricted, however particle size and curing temperature have been demonstrated to impact phosphate geopolymer setting; Zribi *et al.* identified curing temperature as the primary factor, providing around 73.46% of the overall effect on setting time.^86–90^

For porous geopolymers, these chemical differences are important because the same foaming agent may behave differently in alkaline and phosphate-based matrices. Gas release, viscosity development, setting rate and gel chemistry all influence whether pores are retained or lost. Therefore, the activating solution should be selected not only to form a strong geopolymer matrix, but also to match the kinetic requirements of the pore-forming method.

### 2.2. Kinetic and rheological control of foam stability

In porous geopolymer systems, geopolymerisation controls not only the formation of the binding matrix but also the retention of pores during foaming. Pore formation generally occurs while the geopolymer slurry is transforming from a fluid suspension into a viscoelastic and eventually rigid network. The final pore architecture therefore depends on the relative timing of pore generation and matrix hardening. If gas release or air incorporation occurs while the viscosity or yield stress of the slurry is too low, bubbles may rise, coalesce, undergo drainage or escape from the paste, resulting in settlement, heterogeneous macropores and poor foam stability. Conversely, if the matrix stiffens too rapidly, gas expansion may be restricted, leading to incomplete foaming, non-uniform pore distribution or local cracking. Foam stability is therefore governed by the coordination of pore generation, rheological development and geopolymerisation kinetics.

This connection is particularly essential as typical setting time alone does not adequately capture the durability of fresh geopolymer foams. As shown by Dhasindrakrishna *et al.*^78^ for ultra-lightweight geopolymer foam concrete with densities of around 0.43–0.46 g cm^−3^, the stability of the fresh foam was more closely correlated to the initial yield stress and the pace of yield-stress growth than to the first setting time. Their results suggested that the settling in fresh geopolymer foam concrete was due to bubble collapse by the combined influence of drainage, coalescence and ripening. When the initial yield stress was greater than the stability criteria necessary to avoid drainage, the settlement was significantly decreased. This allows the development of bubble retention before the normal initial setting time is attained, provided that the new matrix develops enough rheological resistance.

The significance of early rheological development was also manifested in the hardened pore structure and mechanical performance. Dhasindrakrishna *et al.*^78^ revealed that the more stable geopolymer foam concretes had smaller and more uniform pores whereas the unstable mixtures had bigger and more interconnected pores resulting from bubble collapse and coalescence. The same investigation revealed that the compressive strength was affected by matrix strength and pore features. The foam with finer and more uniform pores showed an improvement of 36% in specific compressive strength for systems with comparable matrix strength. As a result, rheology impacts the stability of new foam as well as the strength of the hardened porous geopolymer by governing pore size distribution and pore uniformity.

Gu *et al.*^91^ investigated the movement of prepared foams in geopolymer paste and compared the pore-formation mechanism with the rheological parameters of the paste, arriving at a similar result. Their investigation demonstrated that the movement of foam was determined by the balance between the external forces on the individual bubbles in the paste, which include buoyancy, capillary force, frictional resistance and drag force. As these forces are regulated by the limiting shear stress of the geopolymer paste, the displacement and deformation of bubbles is directly related to the fresh-state rheology. Gu *et al.*^91^ demonstrated that the absence of limiting shear stress facilitates the flow of foam and the coalescence of pores, whereas too much resistance could result in the breaking of foam or inadequate dispersion. The geopolymer paste with initial limiting shear stress of 22.72 Pa and ultimate limiting shear stress of 87.38 Pa was reported to be more suited for the examined preformed foam system to achieve a homogeneous pore-size distribution. This implies that there must be an appropriate rheological window: the paste must be enough fluid to be mixed with the foam, yet sufficiently strong so as not to allow bubbles to migrate and collapse.

Recent work by Zhang *et al.*^92^ further supports the concept that the design of geopolymer foam should be based on linked control of matrix chemistry, rheology and pore development. They also noted that the behaviour of the geopolymer foams may be described as skeleton–pore dual systems, where the ultimate performance is dominated by the mechanical strength of the matrix skeleton and the pore architecture. The viscosity and the initial setting time were employed as important rheological characteristics in the strategy for the division of the processing space into separate rheological sections. The rheological region was divided in this way in order to correlate process factors such as precursor composition, activator modulus, liquid to solid ratio, curing temperature, foaming agent and surfactant with the pore structure and mechanical strength. Their findings suggested that a lengthy initial setting time may favour pore coarsening, except that a high viscosity might prevent bubble coalescence and ripening. Suitable viscosity was related to a more regular pore structure and high porosity in the medium setting-time areas, on the other hand.

These investigations have shown that the development of porous geopolymers must be regarded as a time-dependent kinetic and rheological process and not only as the addition of a foaming agent. Dissolution, gel formation, viscosity development and setting behaviour are influenced by the precursor type, calcium concentration, activator composition, liquid-to-solid ratio and curing temperature. Then these parameters affect whether bubbles, droplets or detachable templates may be trapped until the matrix is rigid enough to hold the pore design. For example, hydrogen peroxide must be stabilised fast as oxygen evolution might occur quickly in alkaline media. Aluminium and zinc powders may result in a slower evolution of hydrogen but the rate of gas release must be matched to the growth of the matrix stiffness. In microwave assisted foaming the expansion of water vapour must be in synchrony with enough gelation to avoid uncontrolled expansion or breaking. In reactive emulsion templating, the oil droplets must be stable until the matrix is cured enough to retain their form.

Hence, the choice of precursor and activator should be based on not only the attainment of sufficient geopolymerisation and strength, but also an appropriate processing window for the proposed pore-forming approach. A slurry with a very low viscosity may promote bubble escape and pore coalescence whereas a slurry that sets too quickly may hinder uniform expansion. Thus, the best porous geopolymer systems are those where the rates of pore creation and rheological development are matched. This kinetic–rheological approach connects the chemistry of geopolymer formation with the manufacturing techniques mentioned in the subsequent sections and gives a basis to explain why the same foaming agent may induce various pore architectures in different geopolymer matrices.

## 3. Methods of obtaining porous structures in geopolymers

Porous geopolymer can be produced using a variety of fabrication strategies that differ fundamentally in their foaming mechanisms. In the published literature, these techniques are generally grouped into chemical and physical foaming approaches. Chemical routes generate porosity through gas-releasing reactions or the decomposition of reactive species, whereas physical methods rely on templating, phase removal, or structural manipulation. The following subsections provide an overview of the most widely reported techniques and classify them according to the predominant mechanism responsible for pore formation.

### 3.1. Chemical foaming methods

#### 3.1.1. Direct foaming

Direct foaming is one of the most widely applied routes for producing porous geopolymer-based materials because it combines simple processing, low equipment demand and compatibility with conventional casting.^48,93–95^ Nevertheless, direct foaming should not be treated as a single uniform method. It is more appropriately regarded as a group of gas-assisted pore-forming strategies in which the final pore architecture is controlled by the coupled evolution of gas release, slurry viscosity, bubble stability and geopolymer setting. This coupling is critical because the pore structure is formed while the geopolymer matrix is still evolving from a fluid suspension to a rigid network. If gas generation precedes sufficient viscosity build-up, bubbles may rise, merge or collapse, leading to irregular macropores and reduced mechanical reliability. In addition, excessively low viscosity of the system, resulting from slow geopolymerisation kinetics, may also lead to gas loss, as the gas can simply escape from the hardening mass. As a result, the obtained porous material may have an insufficient bulk density. Conversely, if the matrix stiffens too early, gas expansion may be restricted and the resulting porosity may be non-uniform. Therefore, the performance of directly foamed geopolymers is governed not only by the type and amount of blowing agent, but also by the synchronization between gas-generation kinetics and geopolymerisation-induced hardening.^96–100^

Porosity in direct foaming is commonly generated through chemical blowing agents or mechanically introduced air, usually in combination with stabilising additives such as surfactants, oils, thickeners, calcium stearate or solid particles.^50,93–119^ Although unfoamed geopolymer matrices may already contain intrinsic micro- and meso-porosity,^120–122^ deliberately designed cellular structures generally require reactive pore-forming agents, including hydrogen peroxide,^96,123–125^ metallic powders such as aluminium and zinc,^126–128^ silicon-bearing additives,^129–138^ sodium perborate,^139–141^ sodium hypochlorite^142^ and peracetic acid.^143^ The practical advantage of this route is its formulation flexibility: pore volume can be modified through blowing-agent dosage, while pore stability and connectivity can be adjusted through surfactant chemistry, activator composition, solid loading and curing conditions. However, the same flexibility also introduces sensitivity, since small changes in fresh-state rheology or reaction rate may substantially alter pore-size distribution and mechanical performance.^96,99,100,144^

Hydrogen peroxide is the most widely reported chemical foaming agent for geopolymer foams because it decomposes in alkaline media with oxygen release, enabling rapid pore generation.^96,111,123–125^ This makes H_2_O_2_ particularly effective for producing low-density and highly porous materials. Feng *et al.*,^110^ for example, prepared fly ash-based geopolymer foams using type C fly ash, sodium silicate and H_2_O_2_, achieving a density of 0.24 g cm^−3^ and a porosity of 81.2%, while the compressive strength remained 0.67 MPa. These values illustrate a common compromise in peroxide-based direct foaming: high porosity and low density can be achieved, but the resulting mechanical strength may remain limited when rapid oxygen evolution produces heterogeneous pores or weak zones before sufficient matrix stiffening occurs.^96,99,100,110^ Therefore, H_2_O_2_-based foaming is particularly suitable for lightweight and thermal-insulation oriented materials, whereas mechanically demanding applications require additional control over bubble stability and pore refinement.

The need to control peroxide-induced foam instability explains the frequent use of surfactants and other stabilising additives.^97–119,124^ In geopolymer foams, surfactants do more than simply stabilise air bubbles; they influence the processing window by reducing bubble coalescence, modifying liquid drainage and affecting the balance between open and closed porosity. Sodium dodecylbenzene sulphonate (SDBS),^112,124^ sodium dodecyl sulphate (SDS),^113^ sodium lauryl sulphate (SLS),^114^ oleic acid,^115^ octylsulphobetaine (OSB),^116,117^ Lightcrete 02 (ref. 118) and commercial air-entraining agents^119^ have all been investigated for this purpose. Ibrahim *et al.* reported that incorporation of 0.2 wt% SLS reduced the formation of macropores, producing more uniform microstructure of geopolymer.^114^ In contrast, oleic acid associated with interconnected pore networks and irregular openings between adjacent cells, which may be advantageous where permeability is required.^115^ These studies indicate that surfactant selection is a major design variable: depending on the additive used, direct foaming can be shifted towards closed-cell structures suitable for insulation or towards more connected pore networks relevant to transport applications.

Comparative studies further show that the main limitation of direct foaming is not the inability to create porosity, but the difficulty of controlling pore geometry in a deterministic manner. Plantard *et al.* compared direct foaming using H_2_O_2_ with SDS/CTAB surfactants and replication routes using polyurethane or polyethylene templates.^144^ In their direct-foamed samples, pore diameters ranged from 0.7 to 98 µm, while nanopore and micropore volumes were reported as 0.3 mL g^−1^ and 0.22 mL g^−1^, respectively (Fig. 2).^144^ The pore-size distribution also dependent strongly on the foaming agent concentration.^144^ These findings confirm that direct foaming can generate multiscale porosity, but the resulting architecture remains strongly dependent on formulation and processing conditions. Compared with replica or sacrificial-template methods, direct foaming is simpler and more scalable, but it offers less direct control over pore shape, connectivity and spatial arrangement.^144–148^

**Fig. 2:**
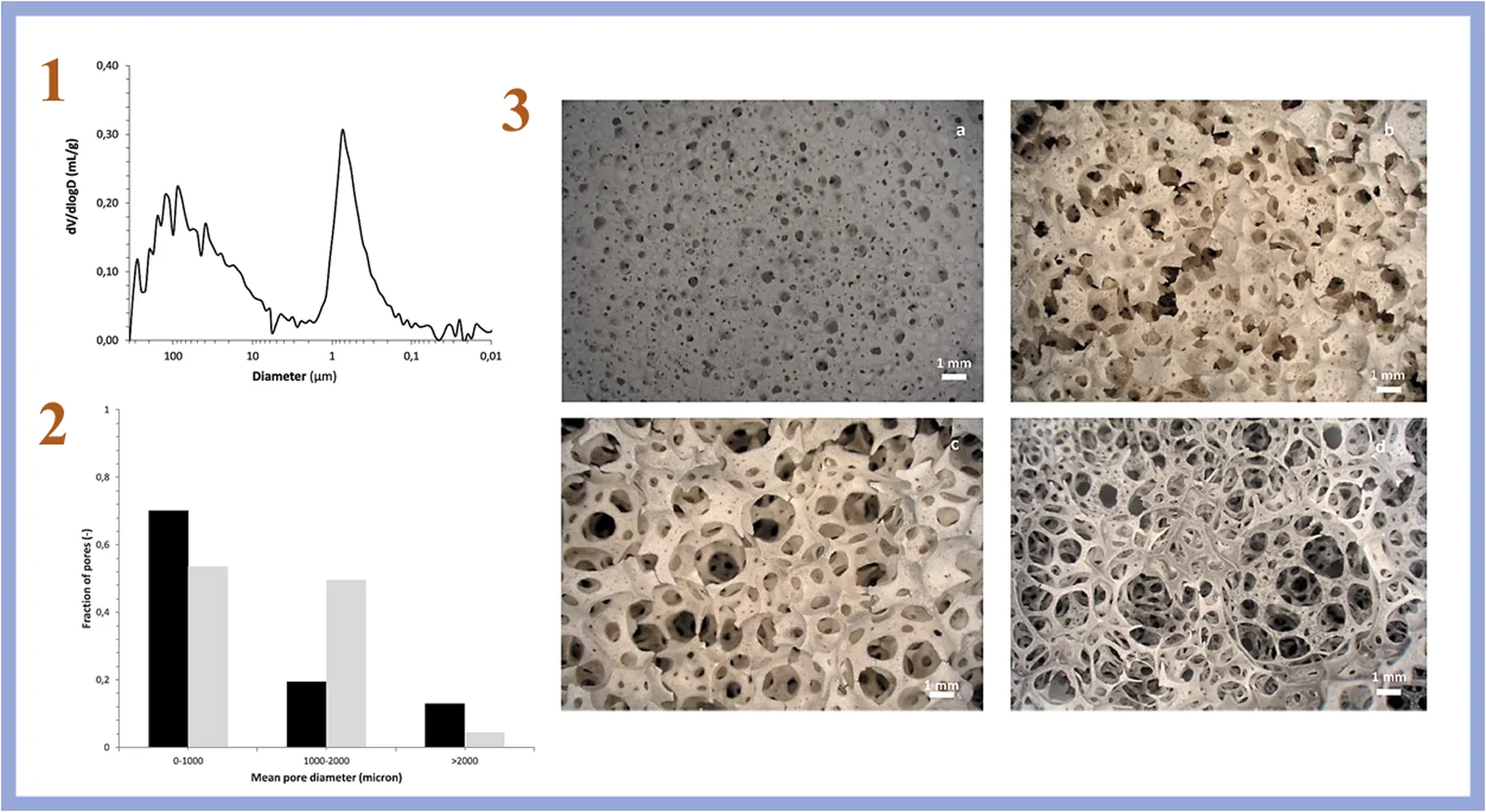
Results of research of Plantard *et al.*: (1) pore size distribution by volume of geopolymer foam (reference foam) synthetized by the direct foaming method; (2) photograph obtained with high-resolution optical microscope-of geopolymer foams elaborated by direct foaming method with different volumes of foaming; (3) variation in pore volumic fraction, obtained by image processing, according to three pore size classes. Results obtained on geopolymer foams synthesised by the direct foaming method agent (a–d): hydrogen peroxide volumes of 0.05, 0.5, 1 and 4 mL. Plantard *et al.*, *Results Mater.*, 2024.^144^

Metallic powders provide another important class of chemical foaming agents. Aluminium and zinc react in alkaline environments with hydrogen evolution and have therefore been widely used to generate pores in geopolymer matrices.^126,127,149–153^ Aluminium is generally more reactive than zinc and can produce larger amounts of hydrogen at lower dosages. Bell and Kriven reported that aluminium reacts more vigorously than zinc and produces approximately 50% more hydrogen on a molar basis.^149^ Yatsenko *et al.* showed that a considerably higher mass of zinc was required to achieve a comparable density reduction,^150^ while Kränzlein *et al.* used zinc at 1.0 wt% of solids compared with aluminium additions not exceeding 0.2 wt%.^151^ These comparisons suggest that aluminium is more efficient for rapid expansion, whereas zinc may offer slower gas evolution. However, high aluminium reactivity can also be problematic if hydrogen release occurs before the geopolymer matrix has developed sufficient stiffness. Moreover, aluminium hydroxide species may form on precursor surfaces and hinder dissolution, thereby delaying strength development.^154^ On the other hand, the use of aluminium has another disadvantage. The reaction between aluminium and alkali is exothermic, releasing a considerable amount of heat; in some cases, the temperature of the system may reach 75–85 °C. Such a sharp temperature increase can also accelerate geopolymerisation compared with its natural progression at ambient temperature. The rapid rise in viscosity creates certain obstacles to the further development of the foamed porous structure, which in turn affects the pore architecture and the properties of the final materials. Thus, the choice between aluminium and zinc should be evaluated not only in terms of theoretical gas yield, but also in relation to reaction rate, pore refinement, matrix hardening and possible interference with geopolymerisation.

Less conventional foaming agents, including sodium perborate, sodium hypochlorite and peracetic acid, have been explored to overcome the rapid and sometimes poorly controlled gas release associated with H_2_O_2_.^141–143^ Sodium perborate can decompose thermally and therefore enables gas evolution to be partially separated from the initial mixing stage.^141,142^ Sodium hypochlorite may release oxygen and carbon dioxide under suitable conditions, providing another route to chemically generated porosity.^142^ Peracetic acid has recently been compared to H_2_O_2_ in metakaolin- and slag-based geopolymer systems.^143^ Bhuyan *et al.* reported that peracetic acid produced substantially lower volumetric expansion than H_2_O_2_, but improved compressive strength (∼7 MPa), which the authors attributed to enhanced precursor dissolution, reduced surface tension and denser gel formation (Fig. 3).^143^ These results suggest that maximum gas release is not always the most desirable objective. In some systems, moderated gas evolution may provide a better balance between porosity, dimensional stability and strength. However, alternative foaming agents must also be assessed in terms of cost, processing complexity, activation requirements and compatibility with the selected precursor-activator system.

**Fig. 3:**
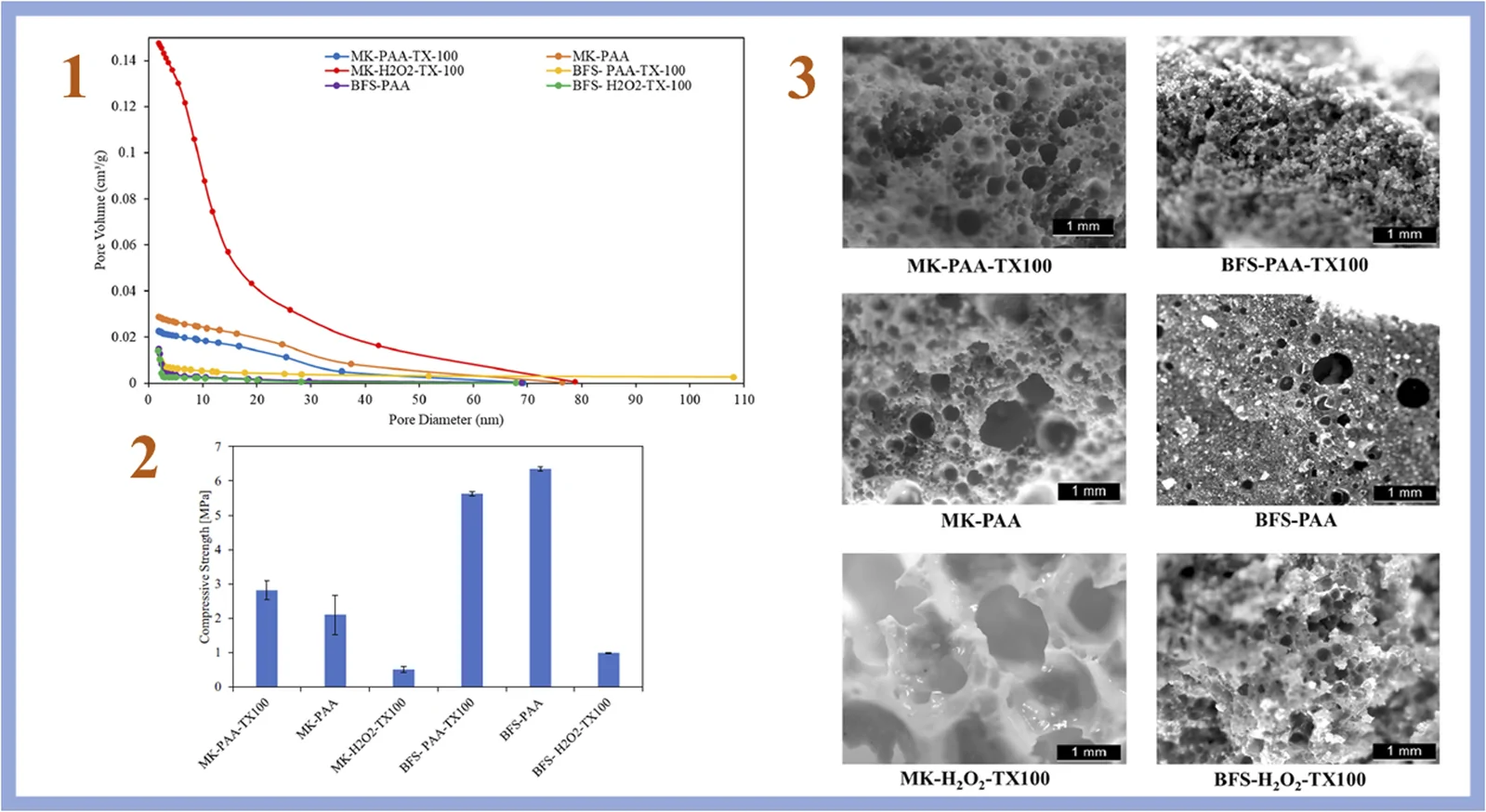
Results of research of Bhuyan *et al.*: (1) cumulative pore volumes of the foams as a function of pore diameter determined by the BJH method; (2) comparison of mechanical strength of BFS and MK foams with PAA or H_2_O_2_ as blowing agents; (3) comparison of optical micrographs of MK and BFS foam based on PAA and H_2_O_2_. Bhuyan *et al.*, *Appl. Clay Sci.*, 2023.^143^

The behaviour of foaming agents becomes even more system-dependent in phosphate-activated or other acid-based geopolymers. These binders differ from conventional alkali-activated aluminosilicate geopolymers because their matrix formation involves Al–O–P, Si–O–P and related phosphate-containing networks.^79–90,155,156^ Therefore, the same foaming agent may not produce the same pore structure across alkaline and phosphate-based systems.

Yang *et al.* compared H_2_O_2_ and aluminium powder in phosphate-based geopolymers prepared from nickel slag and fly ash.^157^ H_2_O_2_ generated rapid oxygen release and produced larger pore volumes and lower dry densities, whereas aluminium produced finer pores and more compact structures through hydrogen generation.^157^ In their study, aluminium-based samples achieved higher long term compressive strength, with the maximum reaching value approximately 3.6 MPa after 7 days, while both foaming agents reduced thermal conductivity to 0.25 W m^−1^ K^−1^ for H_2_O_2_ and 0.19 W m^−1^ K^−1^ for aluminium.^157^ Majdoube *et al.* investigated phosphate activated metakaolin geopolymers using phosphate washing sludge as a CO_2_-releasing solid additive.^158^ Increasing the sludge content reduced density and compressive strength but improved thermal insulation, decreasing thermal conductivity from 0.216 W m^−1^ K^−1^ to 0.076 W m^−1^ K^−1^ at the highest dosage.^158^ These examples demonstrate that binder chemistry, gas-release mechanism and setting behaviour must be considered together when designing directly foamed geopolymer systems.

A particularly noteworthy approach was reported by Roviello *et al.*,^159^ who combined direct foaming with organic–inorganic modification of metakaolin-based geopolymer slurries. Systems containing 10 wt% epoxy resin or dimethylsiloxane oligomers were foamed with 0.03–0.24 wt% silicon powder. Unlike the low-viscosity unmodified slurry, which could not retain the generated gas, the hybrid formulations produced stable foams with apparent densities of 0.25–0.86 g cm^−3^ and open porosities of up to 52% for the epoxy-modified series and 75% for the dimethylsiloxane series. The organic phase also affected pore architecture: epoxy modification promoted a more interconnected network, whereas dimethylsiloxane produced predominantly isolated and more uniformly distributed pores. Compressive strengths of approximately 0.67–11 MPa and thermal conductivities of 0.101–0.105 W m^−1^ K^−1^ were reported.^159^ These results show that hybridisation can improve gas retention and the strength and insulation balance. However, the benefits strongly depend on composition, and excessive expansion increased pore coalescence and reduced mechanical performance.

Taken together, the available studies indicate that direct foaming is the most practical route for producing lightweight geopolymer foams when low density, low thermal conductivity and process simplicity are the primary objectives.^50,93–95,110,144,157,158^ Its main strengths are low equipment demand, compatibility with casting, broad precursor applicability and the ability to generate high porosity. Its main weakness is stochastic bubble formation, sensitivity to fresh-state rheology and the frequent trade-off between density reduction and mechanical strength. Therefore, direct foaming is best suited to insulation and lightweight construction products, while applications requiring well-defined open channels, high permeability or precisely controlled pore geometry may require surfactant-assisted optimization, hybrid processing, replica routes, sacrificial templating or additive manufacturing.^114,115,144–148^

#### 3.1.2. Reactive emulsion templating

Reactive emulsion templating (RET) is a pore-forming strategy in which the porous architecture is generated from a liquid-in-liquid emulsion rather than from gas bubbles. In this method, droplets of an immiscible liquid phase, typically a vegetable oil, are dispersed within an alkaline geopolymer slurry and subsequently removed after setting, leaving behind a pore network that reflects the original emulsion structure.^160,161^ The term “reactive” is particularly important because the dispersed oil phase may undergo saponification under alkaline conditions, producing water-soluble soap molecules and glycerol.^161^ This *in situ* reaction performs two functions simultaneously: it helps stabilise the emulsion during processing and facilitates the later removal of the organic phase. Therefore, RET differs fundamentally from conventional direct foaming, where pore formation is governed by gas evolution, bubble coalescence and foam collapse. In RET, pore size and connectivity are instead controlled mainly by droplet size, emulsion stability, dispersed-phase volume fraction and the ability to remove the organic phase without damaging the hardened matrix.^160,161^

The main advantage of RET is that it provides a more controllable route to open and hierarchical porosity than gas-based foaming. In direct foaming, bubbles form dynamically and may merge or collapse before the geopolymer matrix hardens. By contrast, emulsion droplets can act as relatively stable templates, allowing the pore structure to be tuned through emulsion formulation. This distinction is important for applications in which open porosity, mass transport and accessible surface area are more critical than minimum density. Medpelli *et al.* introduced RET as a route to increase the specific surface area of geopolymers by incorporating triglyceride oils into alkaline geopolymer slurries.^162^ After saponification, curing and extraction in warm water, the highly porous geopolymer frameworks with specific surface area in the range of 55–124 m^2^ g^−1^ were obtained.^162^ These values indicate that RET can produce porous geopolymers with surface areas considerably higher than those typically required for simple thermal insulation, making the method more relevant to adsorption, catalysis and separation applications.

Subsequent studies by Cilla *et al.* further demonstrated that the soap molecules generated during saponification can be exploited as *in situ* surfactants to stabilize the liquid foam before setting.^163,164^ This feature gives RET a distinctive processing advantage: the same chemical reaction that enables template removal also contributes to emulsion and foam stabilisation. However, this advantage also introduces a limitation. The final pore structure becomes dependent not only on the geopolymerisation process, but also on the kinetics of saponification, the stability of the emulsion and the efficiency of organic removal. Inadequate emulsion stability may produce coalesced or non-uniform pores, whereas incomplete extraction of organic species may affect surface chemistry, transport behaviour or long-term durability. Therefore, RET provides greater control than direct foaming, but it also requires more careful formulation and post-processing.

Gossard *et al.* recently extended the RET approach to hierarchically porous geopolymer-based composites designed for fixed-bed applications.^165^ Using an oil-in-water emulsion containing geopolymer precursor and zeolite particles, they obtained monolithic materials with interconnected macropores on the order of several tens of micrometers, together with an intrinsic mesoporous geopolymer matrix.^165^ Nitrogen adsorption–desorption measurements showed type IV isotherms, a BET specific surface area of approximately 120 m^2^ g^−1^ and a total mesopore volume of about 0.55 cm^3^ g^−1^. These results are shown in Fig. 4, are important because they show that RET can combine macroporous transport pathways with mesoporous surface accessibility. Such hierarchical pore systems often lead to irregular macropores and strength loss rather than controlled multiscale architecture.

**Fig. 4:**
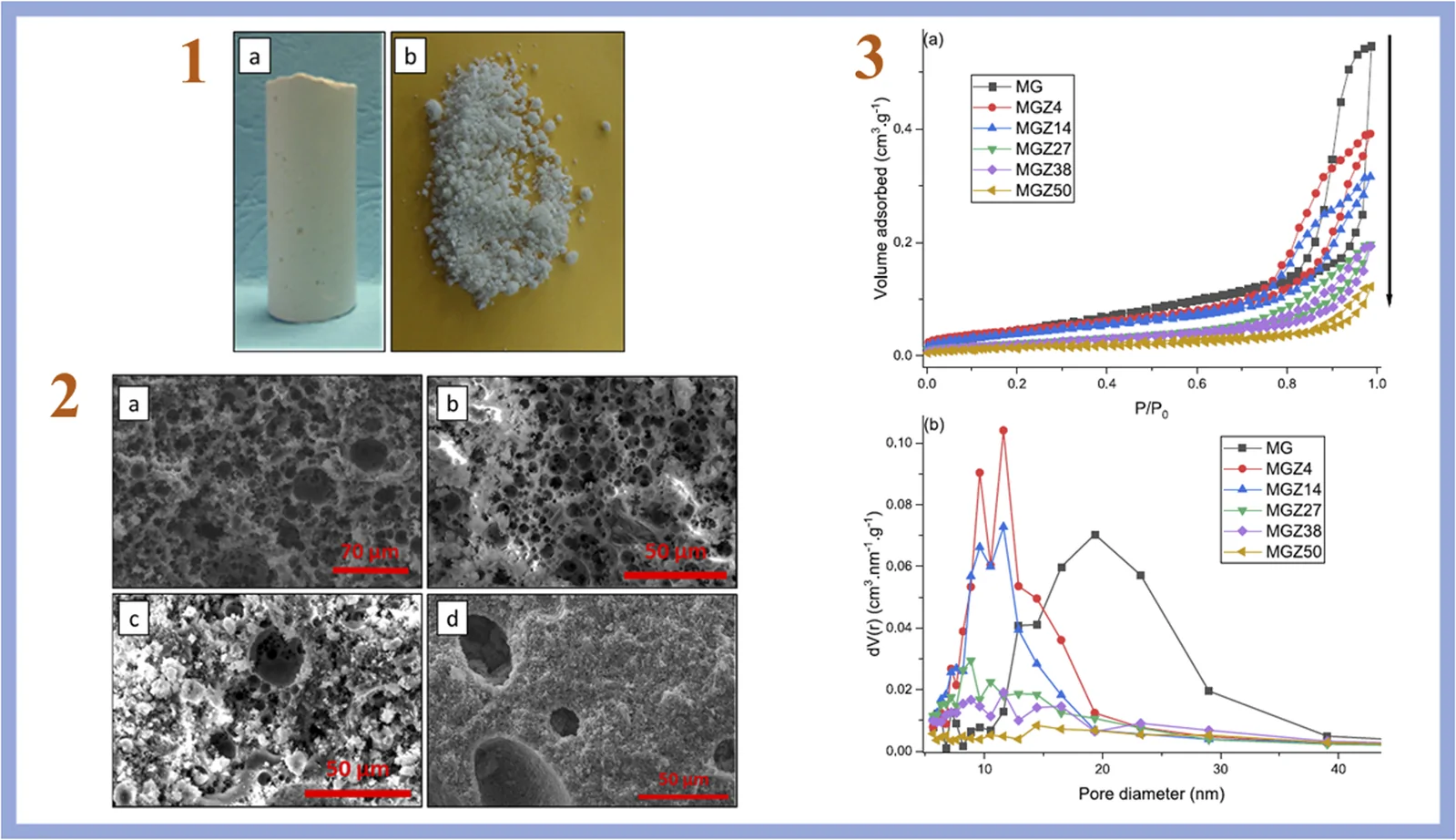
Results of research of Gossard *et al.*: (1) photographs of macroporous geopolymer materials with (a) 27 wt% and (b) 38 wt% of zeolite particles; (2) scanning electron micrographs of macroporous geopolymer materials containing (a) 0 wt%, (b) 14 wt%, (c) 27 wt% and (d) 38 wt% of zeolite particles (MG, MGZ14, MGZ27 and MGZ38, respectively); (3) (a) nitrogen adsorption/desorption isotherms and (b) mesopore size distributions of macroporous geopolymer materials containing 0–50 wt% of zeolite particles. This figure has been adapted/reproduced from ref. 165 with permission from Elsevier under the license number 6240880651331, copyright 2026.

From a processing, structure and property perspectives, RET should therefore be regarded as a specialised route for producing accessible and interconnected pore networks rather than as a general replacement for direct foaming. Its strength lies in the ability to tune pore connectivity, surface area and transport properties through emulsion design. Its weakness lies in processing complexity: emulsion preparation, stabilisation, curing, saponification and organic-phase removal must all be controlled. In addition, the use and subsequent removal of organic oils may raise cost, washing, wastewater and scale-up considerations. These factors make RET less attractive for mass production of low-cost lightweight construction blocks or bulk thermal insulation products. However, for higher-value applications such as adsorption, catalysis, filtration, fixed-bed reactors and functional porous monoliths the additional processing complexity may be justified by the improved pore accessibility and hierarchical structure.^162–165^

Overall, reactive emulsion templating occupies an intermediate position between simple gas-based foaming and more deterministic templating methods. Compared with direct foaming, it offers better control over open and hierarchical porosity, but at the cost of additional formulation and extraction steps.^160–165^ Compared with replica or sacrificial-template methods, it may avoid the need for rigid polymeric templates, but still requires careful management of the organic phase and post-treatment. Consequently, RET is best suited for applications where mass transport, accessible surface area and pore interconnectivity are primary design requirements, whereas direct foaming or lightweight filler incorporation remain more practical for large-volume construction materials where cost, simplicity and scalability dominate.

#### 3.1.3. Microwave-assisted foaming

Microwave-assisted foaming has emerged as a rapid route for producing porous geopolymer-based materials by coupling internal heating, water vaporisation and accelerated geopolymer hardening. Conventional thermal curing of geopolymers commonly requires several hours, and in many cases 24–48 h, depending on precursor composition, activator chemistry and curing temperature. Microwave processing offers a fundamentally different heating mode: instead of transferring heat from the external surface towards the interior, microwave irradiation interacts with polar species, particularly water, and can generate volumetric heating throughout the fresh geopolymer body.^166–171^

The pore-forming mechanism in microwave-assisted foaming (Fig. 5) is primarily associated with rapid heating of pore water and the development of internal vapour pressure.^175^ As the temperature rises, water within the fresh slurry vaporises and expands, generating pores while geopolymerisation and gel hardening are simultaneously accelerated. Therefore, microwave foaming differs from chemically driven direct foaming: porosity is produced by a physical vapour expansion process rather than by the decomposition of a blowing agent such as H_2_O_2_ or by hydrogen release from metallic powders.

**Fig. 5:**
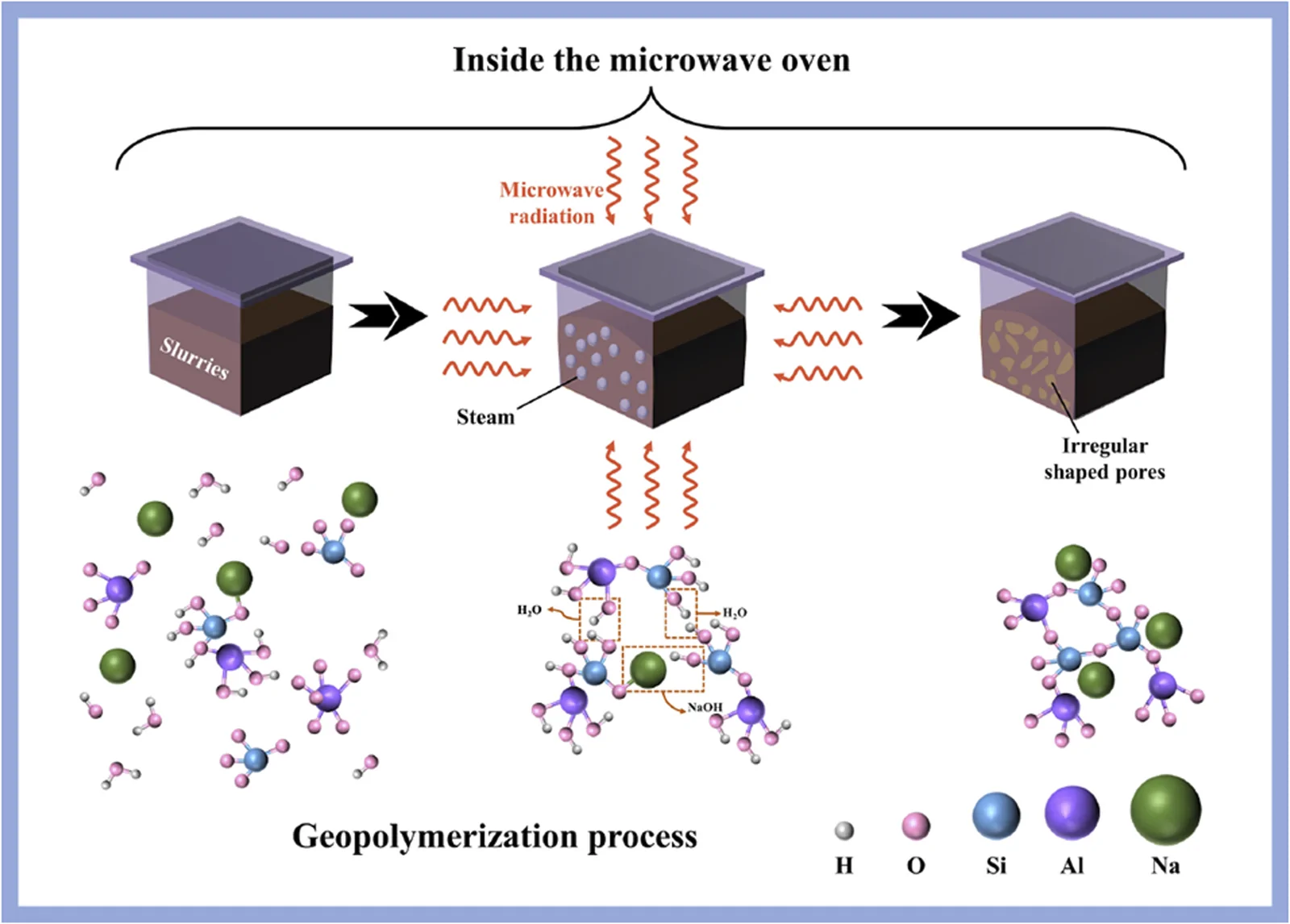
The simplified foaming mechanism and geopolymerisation process of microwave foaming. This figure has been adapted/reproduced from ref. 175 with permission from Elsevier under the license number 6240880819741, copyright 2026.

This makes the process strongly dependent on microwave power, exposure time, water content, sample size and dielectric response of the precursor–activator system. These parameters must be controlled together, because excessive vapour pressure may cause irregular expansion, cracking or non-uniform pore development, whereas insufficient heating may lead to incomplete foaming.

Onutai *et al.* demonstrated the feasibility of this approach by fabricating fly ash-based porous geopolymers using a domestic microwave oven.^172^ In their study, fly ash rich in SiO_2_ and Al_2_O_3_ was activated with sodium silicate and NaOH solutions of different molarities, and the prepared slurry was exposed to microwave irradiation for only one minute at power levels between 200 and 850 W.^172^ This example illustrates the major processing advantage of microwave foaming: pore formation and early hardening can occur on a minute scale rather than over many hours. However, the use of a domestic microwave system also highlights a broader challenge for scale-up, because field distribution, sample geometry and local overheating may strongly influence pore uniformity.

Ultra-lightweight geopolymer foam aggregates were prepared from coal fly ash by microwave curing with sodium hydroxide and sodium silicate as alkaline activators by Hanif *et al.*^173^ The 5 min microwave-oven curing regime expedited hardening and pore formation resulting in aggregates with extremely porous internal morphology and bulk densities about 50% lower than the ASTM maximum limit of 0.88 g cm^−3^ for structural lightweight aggregate (Fig. 6).^173^ The loss of compressive strength due to increased porosity was compensated by sufficient impact resistance and water absorption behaviour of the aggregates for lightweight structural and insulation-oriented applications.^173^ This indicates that microwave-assisted foaming may be especially beneficial when quick generation of lightweight aggregates is preferred over very regular pore geometry.

**Fig. 6:**
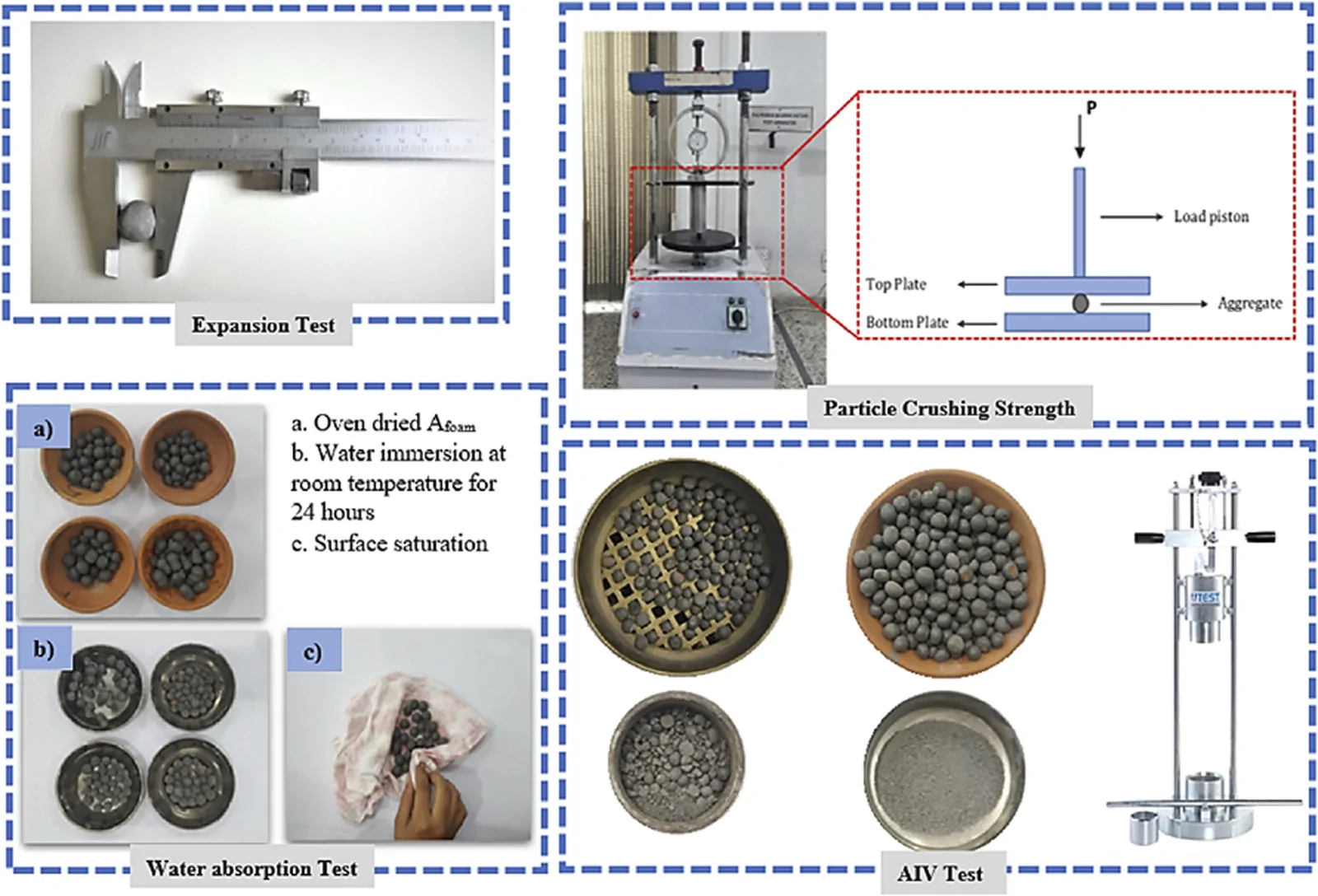
Pictorial demonstration of procedure for different tests performed on lightweight fly ash-based geopolymers, Hanif *et al.*, *J. King Saud Univ., Eng. Sci.*, 2023.^173^

Li *et al.*^174^ reported a quick microwave foaming process for alkali-activated foams from self-igniting coal gangue. Pore water was rapidly heated to boiling by microwave irradiation at around 700 W, leading to vapour-driven expansion, whereas geopolymer gel formation happened virtually immediately.^174^ The materials obtained had overall porosities in the range 74–84 vol% and low bulk densities.^174^ Of note, Li *et al.* discovered irregular pore geometries but better compressive strength at the same porosity for microwave foamed materials compared to directly foamed samples, ascribed to improved precursor reactivity and decreased sodium silicate phases.^174^ Microwave foaming may not provide the most uniform pores, but quick heating can change reaction kinetics and matrix formation, somewhat compensating for the irregularity in morphology.

Zheng *et al.* further developed microwave foaming for highly porous metakaolin-based geopolymers.^175^ The rapid volumetric heating resulted in the vaporisation of water in the slurry and expansion of the foam, while increasing the geopolymerisation.^175^ This dual mechanism allowed for the fabrication of porous materials with overall porosity between 74 and 84 vol% and much less processing time than the replica and reactive emulsion templating procedures.^175^ The foams obtained had irregular pore shape instead of the more spherical pores usually associated with traditional chemical foaming, yet they showed competitive mechanical behaviour at comparable porosity levels.^175^ These data support the idea that microwave foaming is predominantly a quick processing approach rather than a means for controlling the pore-geometry precisely.

Microwave-assisted foaming provides a number of potential advantages compared to direct chemical foaming. First, it eliminates or minimises the requirement for chemical blowing agents, which may reduce formulation complexity and avoid any by-products related with gas producing additives. Secondly, the microwave power and the exposure period are externally tuneable, thereby allowing a more direct control of the reaction than reactions driven by blowing agent breakdown. Third, fast heating may facilitate precursor dissolution, gel formation and early strength development, which may enhance the strength and porosity balance in chosen systems.^174,175^ However, these benefits ought to be read with some caution. Microwave foaming does not guarantee uniform porosity, as local variations in water content, dielectric characteristics and sample thickness might lead to internal heating gradients. These gradients may cause non-uniform porosity, localised dilation or cracking, especially after scaling up from tiny lab specimens to big building elements.

Microwave-assisted foaming has the most promising applications in lightweight aggregates, insulating materials and fast-produced low-density geopolymer components where short processing time is a benefit.^172–175^ In its current form it is less appropriate for applications necessitating highly organised open channels, small pore-size distributions or carefully specified flow routes, such as filtration membranes, catalytic supports or architected scaffolds. For these applications, replica processing, sacrificial templating, reactive emulsion templating or additive manufacturing may offer greater control over pore connectivity and shape. Therefore, future research of microwave foaming should focus on managing the internal temperature distribution, water content, specimen size and curing processes, since these are the elements that decide whether fast vapour expansion results in usable porosity or uncontrolled flaws.

In summary, microwave-assisted foaming is between the direct foaming and deterministic templating techniques. Similar to direct foaming, it may produce high porosity through a dynamic pore-forming event, however unlike traditional chemical foaming, it is driven by physical water vaporisation instead of gas-releasing chemical processes. Its greatest strength is its rapidity, with pore development and hardness occurring within minutes in published tests.^172,173^ The major restriction is the pore irregularity and scale-up sensitivity, especially when the microwave penetration depth, heating uniformity and moisture distribution are difficult to manage.^174,175^ Therefore microwave-assisted foaming can be considered as a viable fast-processing way for lightweight geopolymers but not yet as a general alternative for templating or additive manufacturing methods where precise pore architecture is needed.

### 3.2. Physical foaming methods

#### 3.2.1. Embedding lightweight fillers

Another way to get low-density geopolymer composites is to use lightweight or porous fillers. Here porosity is introduced by an existing solid phase and not by dynamic foaming. This technique employs a geopolymer slurry containing hollow or porous particles, leading to materials similar to syntactic foams.^176–178^ This method is fundamentally different from direct chemical foaming or microwave aided expansion. In gas-based foaming techniques, the pore shape is dependent on gas production, bubble stability and setting kinetics. On the other hand, in filler-based systems, the principal pore properties are set by the filler type, particle size distribution, volume fraction, shell strength and compatibility with the geopolymer matrix. Thus, the use of lightweight fillers may provide a more repeatable way of obtaining low density materials, especially where a moderate porosity and mechanical dependability are essential.

Geopolymer composites have been studied with various lightweight and porous fillers, including fly ash-based particles,^179^ hollow glass microspheres,^180–182^ coal gangue,^183^ ceramic fillers,^184–187^ cenospheres,^188^ expanded polystyrene^189^ and expanded vermiculite.^190^ The fillers have different densities, particle shape, stiffness, water absorption, thermal stability and surface chemistry. Thus, their influence on geopolymer performance cannot be assessed just as density decrease. For example, hollow fillers can decrease heat conductivity by the introduction of closed air-filled voids, but excessive filler content may impede load transmission if the filler shell is brittle or if the filler–matrix interface is inadequately bonded. Similarly, porous mineral fillers may increase thermal insulation and weight reduction, but their water absorption may modify the effective water-to-solid ratio, workability and geopolymerisation kinetics.

The major benefit of filler induced porosity is the possibility to get a suitable strength and density balance. These merged pores are not huge as they are not formed from unstable gas bubbles but from discrete particles related to the pore shape. This leads to a procedure that is less sensitive to the collapse of the foam than direct foaming and less dependent on the creation of internal vapour pressure than microwave processing. However, this benefit depends on the filler remaining intact during mixing, well distributed throughout the slurry and bonding enough with the geopolymer matrix. If the filler is crushed it segregates or generates weak interfacial zones and the predicted strength gain is lost. Therefore, the key design characteristics for this approach are filler volume percentage, particle grading, mechanical integrity, surface compatibility and mixing technique.

Fly ash cenospheres, which are hollow and spherical, and also an industrial by-product, are one of the most important fillers for lightweight geopolymer composites. Low density geopolymer foams were synthesised using fly ash cenospheres as pore-forming inclusions by Peng *et al.*^191^ The presence of cenospheres lowered the dry bulk density to 0.69 g cm^−3^ with compressive strength of around 5 MPa.^191^ This study highlights the benefit of syntactic geopolymer foams: in comparison with highly expanded gas foamed systems, filler-based composites may preserve meaningful mechanical strength at very low density, since the pore structure is physically restricted in hollow particles. At the same time, the study also reveals that the thermal behaviour of cenosphere-containing systems should be carefully studied, as the inclusion of filler might affect heat transmission and reaction enthalpy at elevated temperatures (Fig. 7).^191^

**Fig. 7:**
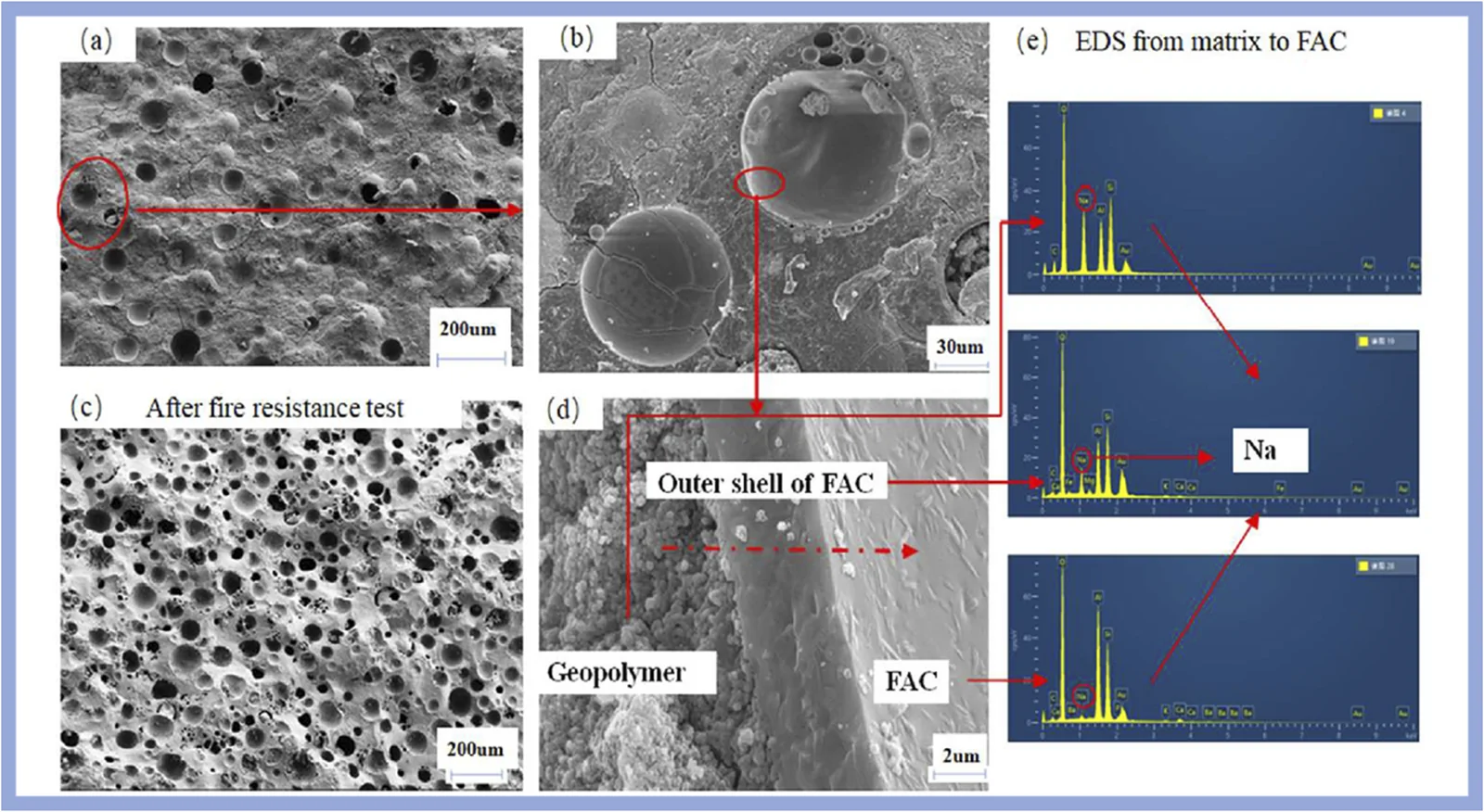
Pictures and energy dispersive spectroscopy (EDS) of geopolymer (50% fly ash cenospheres (FACs) addition): (a), (b), (d) pictures of geopolymer with 50% FAC addition before fire resistance test; (c) pictures of geopolymer with 50% FAC addition after fire resistance test; (e) EDS of selected geopolymer specimens. This figure has been adapted/reproduced from ref. 191 with permission from Elsevier under the license number 6240881031494, copyright 2026.

With respect to construction, the use of lightweight filler is particularly appealing since it can be used with traditional mixing and casting procedures and does not require a further foaming reaction, emulsion preparation, template removal or digital printing process. This offers the approach a practical benefit for light blocks, panels, insulation elements and fire-resistant components. Moreover, many of the fillers such as cenospheres, coal gangue, expanded vermiculite or fly ash-derived materials are waste-derived or mineral-based, which is in accordance with the low-carbon and circular-economy aims of geopolymer technology.^179,183,188,190,192^ However, the sustainability advantage relies on the availability of the filler, the distance it has to be transported, the processing needs and if the filler enhances performance enough to justify its usage.

The fundamental disadvantage of this method is that the porosity created is typically closed or poorly linked. So, this is good for thermal insulation because closed air-filled holes restrict heat transmission but less good for situations where fluid flow, adsorption or catalytic accessibility are required.

For such applications, reactive emulsion templating, replica processing, sacrificial fillers or additive manufacturing may be more suitable, as they may generate linked open-pore networks. Therefore, the embedding of lightweight fillers should be seen as a construction-oriented technique for enhancing density, thermal performance and mechanical dependability, rather than as a universal solution for all the porous geopolymer applications.

Overall, the use of lightweight fillers is one of the most feasible ways to prepare geopolymer based syntactic foams. Advantages are repeatability, compatibility with existing building techniques, less dependency on foam stability and the potential of obtaining a desirable strength–density balance.^176–192^ The limitations are the necessity for acceptable pre-formed fillers, probable breakage of the filler during mixing, interfacial bonding problems, water absorption and inadequate control of open interconnected porosity. Therefore, this technology is best suited for lightweight construction composites, insulating panels and fire-resistant building components while filtration, adsorption and catalytic applications often demand more linked pore designs.

#### 3.2.2. Additive manufacturing

Additive manufacturing (AM) is a fundamentally new approach to the production of porous geopolymer-based materials, as it enables the pore architecture to be defined digitally prior to fabrication. In typical direct foaming, pore size, shape and connectivity are controlled by gas development, bubble coalescence, slurry viscosity and setting kinetics. In replica or sacrificial-template approaches, the ultimate porosity is determined by a physical template or detachable phase. Instead, in AM, macroporosity may be induced by programmable toolpaths, lattice geometries, regulated filament spacing or powder-bed architecture.^193–204^ Hence, AM should be seen not only as a forming technique, but also as a process to develop porosity from a stochastic material characteristic to a designed structural parameter.

This difference is crucial as many applications demand more than high total porosity. Different pore size, connectivity, orientation and mechanical stress routes are required for thermal insulation, filtration, catalysis, acoustic dampening, lightweight structural elements and bespoke building components. AM provides the option of manufacturing periodic lattices, graded porosity, directed channels and complicated geometries that are difficult or impossible to achieve by direct foaming or simple casting.^193–204^ In this regard, AM gives the highest degree of architectural control among the fabrication routes addressed in this analysis. However, this control is obtained at the expense of tougher requirements for ink composition, printing stability, curing control, manufacturing time and equipment.

Among the Additive Manufacturing (AM) techniques, Direct Ink Writing (DIW) is of special interest for geopolymer systems since it allows the paste-like precursor to be extruded *via* a nozzle and the material to be deposited layer-by-layer. The major problem is that geopolymer inks are reactive suspensions and not inert printable pastes. During printing, dissolution, polycondensation and gelation occur continually leading to time-dependent variations of viscosity, yield stress and thixotropy. This means a printable geopolymer ink has to fulfil conflicting demands: it has to be fluid enough to be extruded through the nozzle, stiff enough to keep its shape after deposition, thixotropic enough to recover structure between layers and stable enough to remain printable during the needed open time. This rheological conflict is the key bottleneck of geopolymer AM.^195–199,205^

Different additions have been studied in order to adjust the rheological behaviour of the geopolymer inks such graphene oxide (GO),^195,197^ Acti-Gel® 208 and cellulose-based additives,^199^ poly(acrylic acid) sodium salt (PAA),^196^ and polyethylene glycol (PEG).^198,199^ These additives may impact plasticity, yield stress, buildability and form retention. But they may also affect setting kinetics, porosity, mechanical strength and long-term durability. Thus, rheological modifiers should be viewed not just as processing aids but as part of the structure building chemistry of printed geopolymers. A good printing formulation may not give the greatest hardened performance while a formulation with high final strength may not have adequate open time for printing. This trade-off is one of the major reasons why AM of geopolymers is still less advanced than traditional casting or moulding.

Franchin *et al.* showed the possibility of DIW for the production of geopolymer lattices with controlled spacing and planned porosity architecture.^205^ The importance of their work lies in the demonstration that AM can decouple total porosity from random pore generation, thereby enabling the pore size and interconnectivity to be controlled by the printed geometry rather than by gas evolution or template removal.^205^ The compressive strength–porosity relation published for printed lattices shows that mechanical performance may be tuned by architectural design, such as strut spacing, lattice shape and load-bearing channel (Fig. 8).^205^ This is an important benefit over direct foaming where an increase in porosity often leads to a less predictable decrease of strength due to pore coalescence and uneven distribution of the pores.

**Fig. 8:**
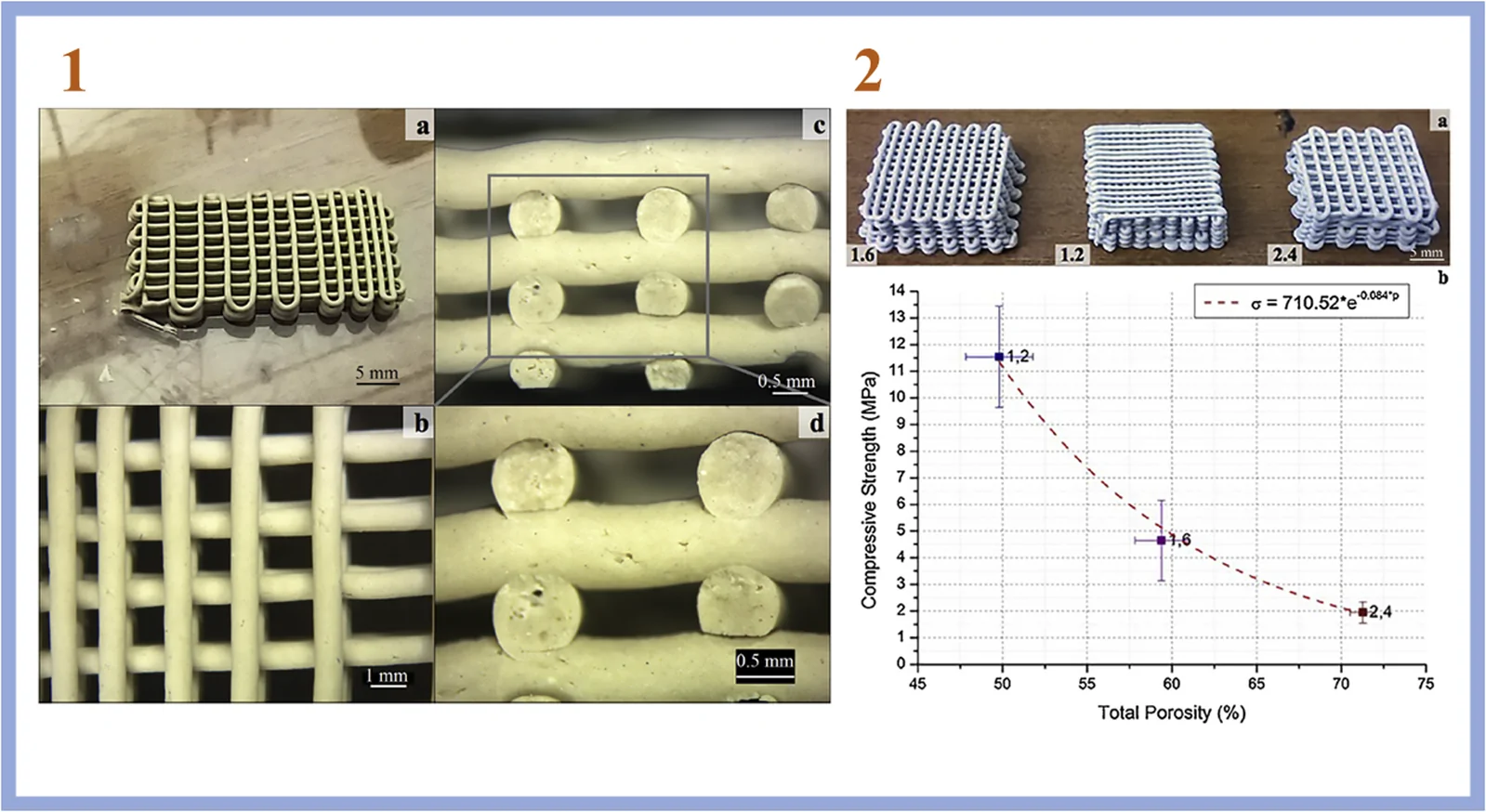
Results of research of Franchin *et al.*: (1) photographs of a lattice with a gradient in spacing: (a) overview, (b) top view, (c) and (d) side views; (2) (a) lattices with (I) 1.6 mm, (II) 1.2 mm and (III) 2.4 mm spanning distance; (b) compressive strength *vs.* porosity plot for the three types of lattices. This figure has been adapted/reproduced from ref. 205 with permission from Elsevier under the license number 6240881339123, copyright 2026.

Powder-based additive manufacturing offers an alternative way to porous geopolymer bodies. Xia and Sanjayan have developed porous sodium-based geopolymer structures utilising a powder-bed printing technique in which geopolymer particles are selectively bound by an organic binder dispensed by a printing head.^206^ Schematic illustration of powder-bed printing process is depicted in Fig. 9. The components obtained exhibited an apparent porosity of around 57 vol% and a compressive strength of about 0.9 MPa.^206^ The porosity was rather homogeneous and was due mostly to interparticle gaps rather than from intended lattice voids.^206^ This illustrates a key difference in AM subtypes. Although it is possible to print uniformly porous materials with powder-based printing, the geometry freedom for the construction of huge ordered channels or load-bearing lattices is lower than that of DIW. The comparatively low strength also implies that post-processing such as sodium silicate infiltration would be essential when improved mechanical performance is desired.^206^

**Fig. 9:**
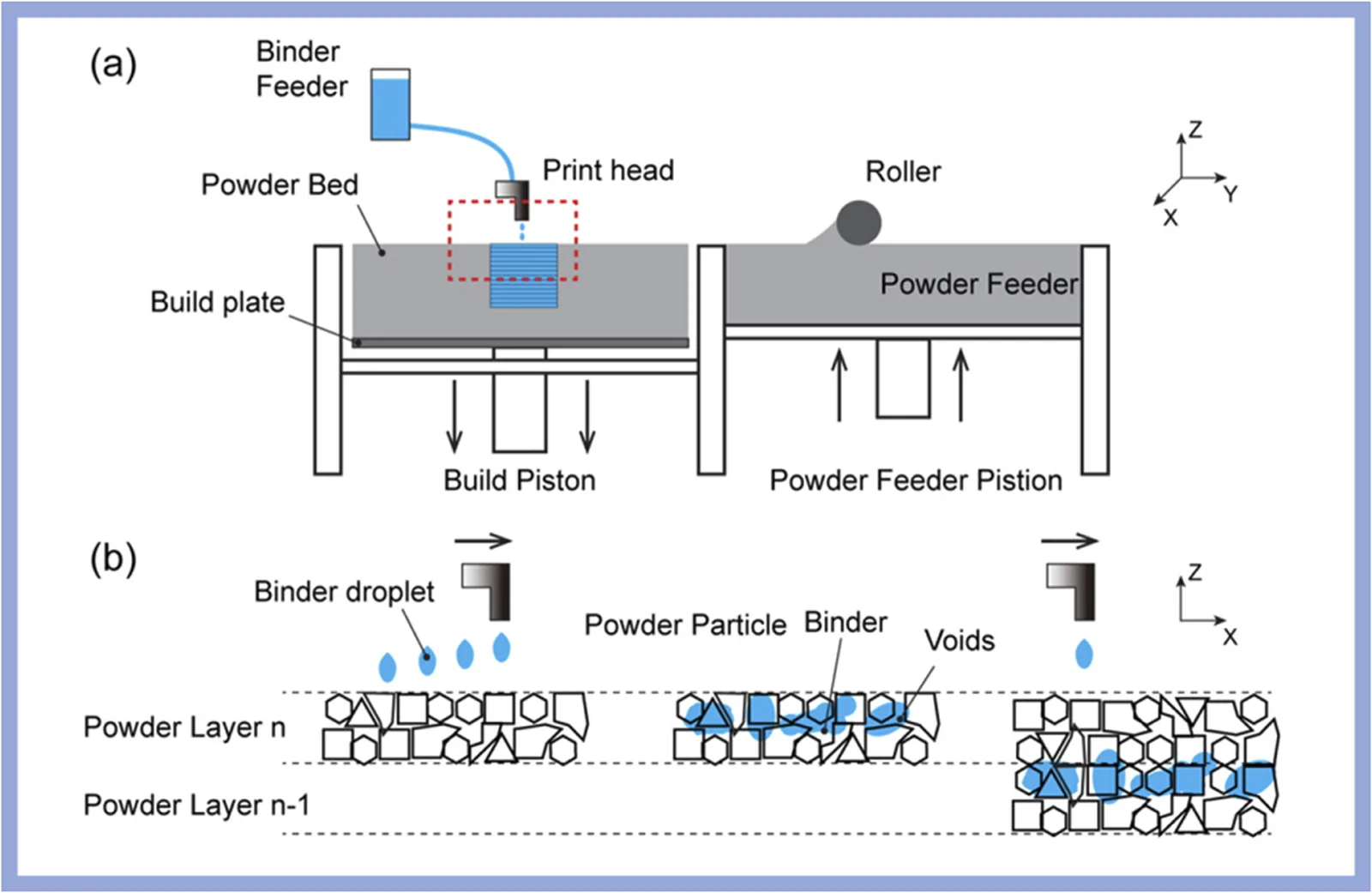
Schematic illustrations of the 3DP process: (a) 3DP inkjet printing system, (b) enlargement of the area in red rectangle: powder/binder interaction between adjacent layers. This figure has been adapted/reproduced from ref. 206 with permission from Elsevier under the license number 6240881484965, copyright 2026.

Zhong *et al.* established an extrusion-based AM method for the geopolymer/graphene oxide composites and shown that the rheology control helped by plasticiser could enable geopolymers with varied alkali cations and Si/Al ratios to be appropriate for 3D printing.^196^ Compared with powder-based techniques, which may need binder removal or infiltration, extrusion-based printing may yield denser structures and, in this case, compressive strengths of 60 to 110 MPa were observed (Fig. 10).^196^ These findings indicate that AM does not inherently imply weak porous materials, and excellent mechanical performance may be kept when the printed architecture and matrix composition are adjusted. However, the comparison with powder-based printing also reveals that performance is largely dependent on the selected AM method, degree of planned porosity, matrix densification and post-processing.

**Fig. 10:**
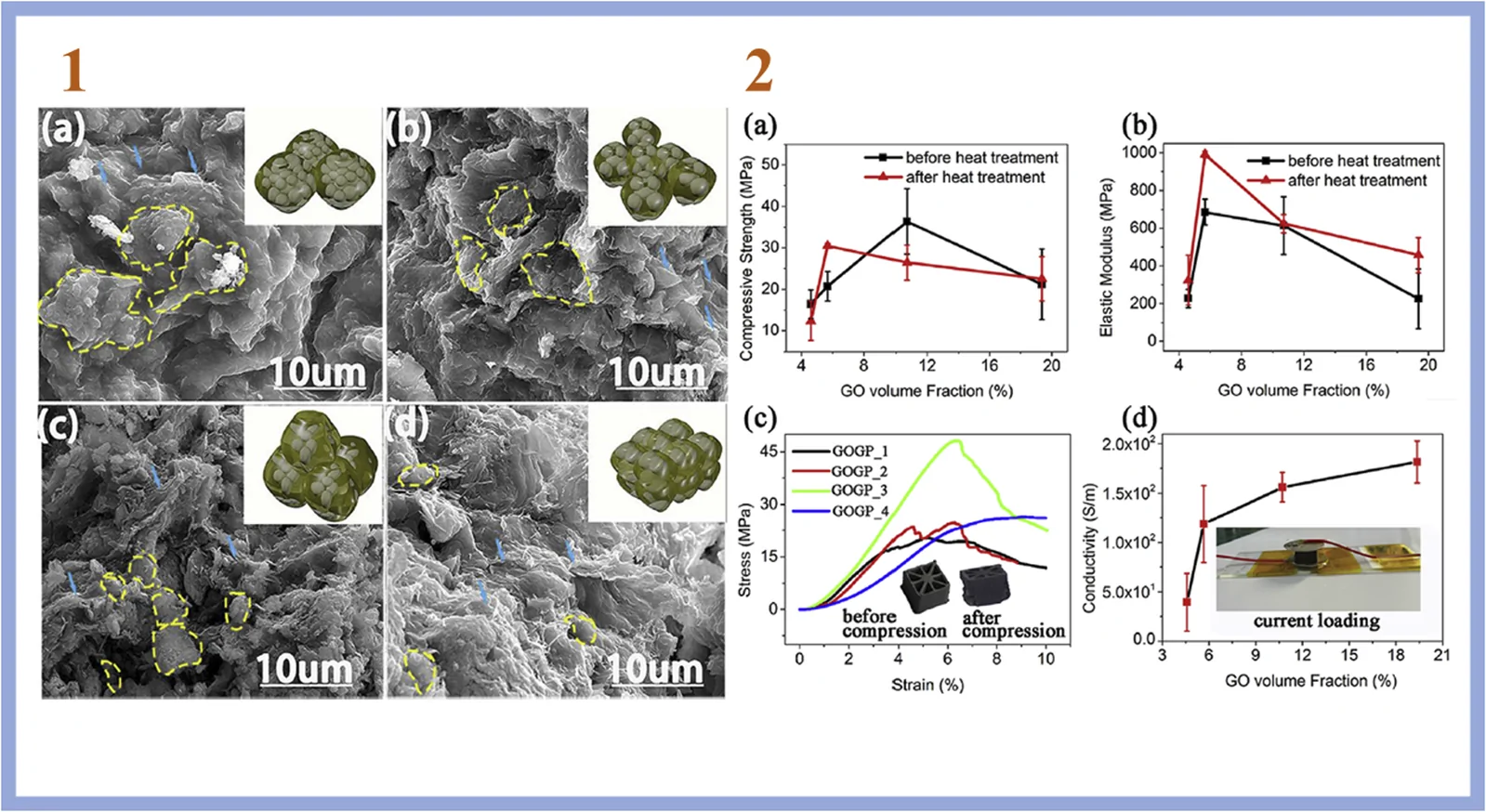
Results of research of Zhong *et al.*: (1) SEM images (a–d) and the corresponding models for the illustration of hydrated geopolymer particles encapsulation by graphene oxide (GO) layers. As the loading of GO in nanocomposite increased, the size of the particle agglomerate (marked by dotted-line circles) that encapsulated within GO films decrease; (2) mechanical properties (a–c) and electrical conductivity (d). The inset of (c) are the images for the 3D printing geopolymer/GO samples before and after compression test. This figure has been adapted/reproduced from ref. 196 with permission from Elsevier under the license number 6240890153020, copyright 2026.

From a cost and scalability point of view AM is a technologically distinct approach compared with direct foaming, light weight filler integration and casting. Direct foaming and filler-based techniques are more compatible with current building manufacturing lines, can be scaled up by mixing and casting and are typically more suited for high-volume low-cost products, *e.g.*, blocks, panels and insulating components. In contrast, AM has slower build rates, tighter control of material and more costly equipment and is more sensitive to printing settings. It is consequently unlikely to have a short-term utility in replacing typical low-cost insulating blocks. Instead, AM is more viable for high-value or bespoke components where geometric complexity confers functional benefits, *e.g.*, graded acoustic panels, tailored filters, catalytic supports, lightweight lattice elements, repair components or architected fire-protective structures.

Curing and dimensional stability are also required for effective use of geopolymer AM. Since the geopolymerisation process continues during setting, drying and perhaps heat treatment of the printed item, it is important that the geometry of the printed object is maintained. Shrinkage, interlayer weakness, anisotropic strength, and poor bonding between neighbouring filaments may cause performance degradation, especially in larger components. In tiny laboratory specimens these problems are not so obvious, but crucial during scale-up. Therefore, future development should focus on printability as well as interlayer bonding, curing procedures, long term durability, moisture resistance and standardised testing of printed porous geopolymers.

Of the fabrication approaches for porous geopolymers, additive manufacturing offers the highest degree of control of the pore-architecture but is also one of the most demanding in terms of formulation and processing. The key advantage is the capability to produce application specific, non-stochastic geometries with regulated pore size, orientation and connectivity.^195,204,207^ The key limits are the rheological complexity, the restricted open time, the slower production rate, the equipment expense, the curing sensitivity and the scale-up issues.^195–199,205,208^ Thus, AM is best suited for cases where geometric control is beneficial beyond only density reduction. Direct foaming and lightweight filler integration are still more viable at present for bulk thermal insulation or low-cost lightweight building goods. AM offers an unprecedented amount of design freedom not easily offered by other technologies for bespoke components, flow-optimized filters, architected scaffolds and graded multifunctional pieces.

#### 3.2.3. Replication method

The replica technique is a template-assisted fabrication process, mainly for the production of open-cell and highly linked porous geopolymer structures. In contrast to direct foaming, where the development of pores depends on the release of gas and the stability of the bubbles, the replica technique employs a ready-made porous template, normally a polymeric sponge, which is transferred to the network of the geopolymer or the geopolymer derived ceramic. First the template is impregnated with a geopolymer slurry. The surplus slurry is removed and the coated structure is cured or dried. The organic template is removed by thermal treatment or other removal method. Consequently, the resulting pore architecture is mostly controlled by the pore size, connectivity and shape of the sacrificial template rather than random bubble generation.

This technique provides the replica approach a unique advantage when open and continuous pore networks are required. Direct foaming results in a possible partial pore interconnection and it is heavily influenced by surfactant type, bubble coalescence and setting kinetics. In contrast, the polymeric sponge template already has a linked cellular network that is replicated by the geopolymer slurry during impregnation and hardening. Replica processing is thus of interest primarily in applications for fluid movement such as filtration, catalyst supports, membranes, filtration of molten metals and permeable ceramic-like structures. This benefit is nevertheless at the cost of added processing complexity, where effective manufacturing needs appropriate slurry infiltration and uniform coating of template struts, regulated drying and full template removal without excessive cracking or shrinking.

Kovářík *et al.*^145^ showed the application of the replica technique for synthesis of open-cell ceramic foams using geopolymer precursor and polyurethane sponge as sacrificial template. The geopolymer slurry was prepared from calcined kaolin clay, precipitated silica and potassium silicate solution with chamotte filler added to prevent cracking during heat treatment.^145^ The polyurethane template contained 7–15 pores per inch and bulk density of 0.019–0.022 g cm^−3^.^145^ The samples were impregnated, dried and sintered at 1100–1300 °C for 4 h. The compressive strength rose from 0.15 MPa at 1100 °C to 0.42 MPa at 1200 °C and 0.85 MPa at 1300 °C. The open porosity was 87.96 vol%.^145^ These results highlight the major strength-porosity trade-off of the replica route: very high open porosity and permeability are attained but the mechanical strength is restricted, as the load-bearing phase comprises of thin linked struts.

Bai *et al.*^146^ also generated open-cell cordierite-based geopolymer/polyurethane foams, employing a 40 PPI polyurethane foam template (Fig. 11). The impregnated samples were cured at 75 °C for 24 h, exposed to NH_4_Cl ion exchange, dried and sintered at 1200 °C for 2 h.^146^ Sintering raised the compressive strength from ∼0.21 MPa to 0.47 MPa with the total porosity remaining almost same, falling slightly from ∼89 vol% to 88.64 vol%.^146^ This discovery is crucial as it indicates that the mechanical strength may be improved post treatment without a significant loss of the open porosity inherited from the template. In the meanwhile, the requirement for ion exchange, drying and high temperature sintering underlines the processing load of replica generated geopolymer foams.

**Fig. 11:**
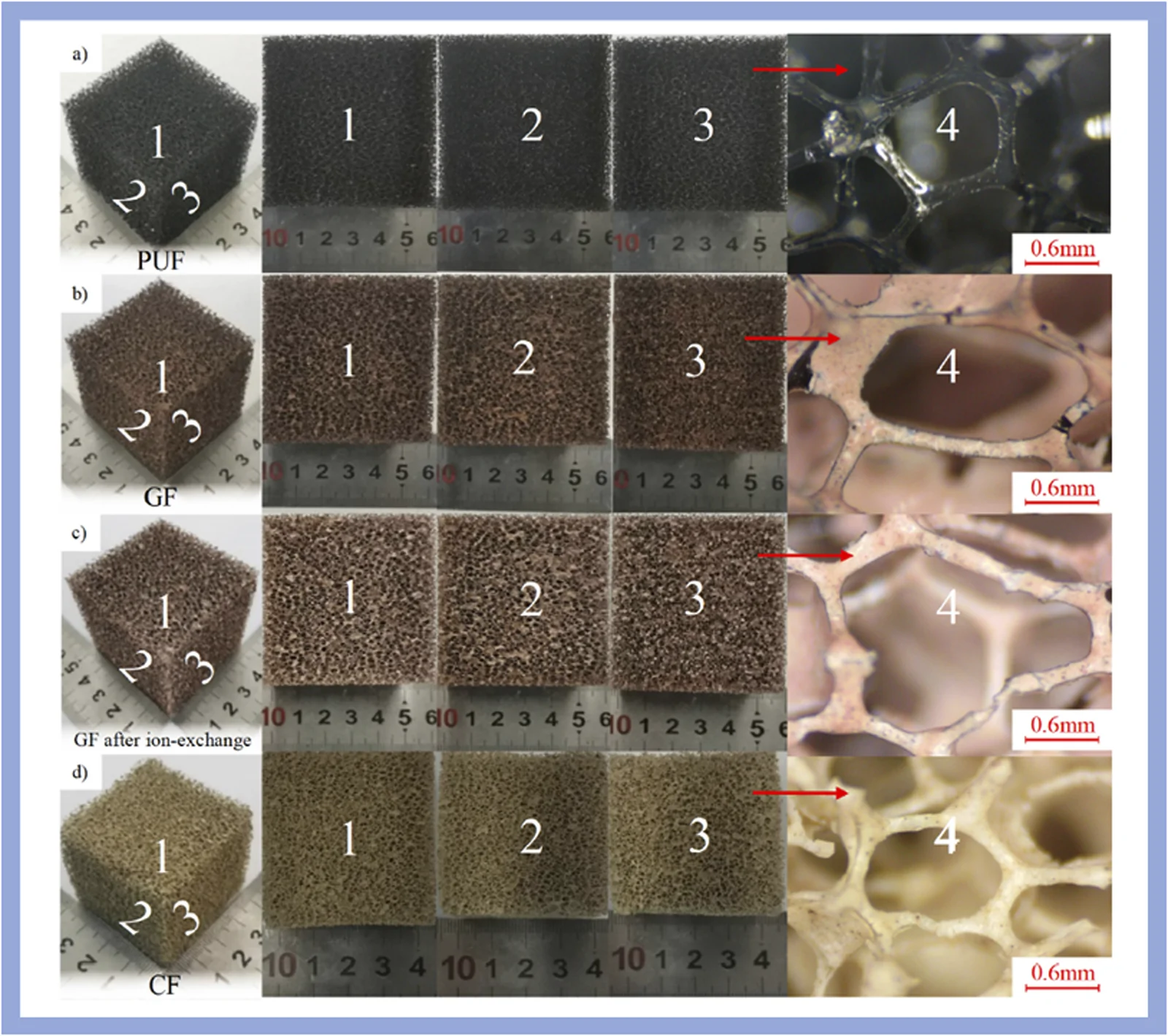
Macroscopic images (1–3) and optical stereoscopic micrographs of the (4) surface of the polyurethane foam (a), the foam after geopolymer slurry immersion (b), the geopolymer foam after ion-exchange treatment (c), and the ceramic foam after sintering (d). This figure has been adapted/reproduced from ref. 146 with permission from Elsevier under the license number 6240890326075, copyright 2026.

The replica approach has greater control of the pore connectivity than direct foaming, but less flexibility than additive manufacturing. The pore network is not digitally constructed, but inherited from the sacrificial template. Therefore, the feasible architecture is limited by the available template geometry, pore size and mechanical stability. Polyurethane sponges are desirable because they are affordable, highly interconnected and commercially accessible with various pore densities. However, polymer template burnout causes energy consumption, gaseous emissions, potential residue formation and shrinkage hazards. These considerations are of particular relevance in the assessment of the approach from the perspective of low-carbon building, since the environmental advantage of a geopolymer matrix might be somewhat diminished by the high-temperature template removal or ceramic conversion phases.

The technique also has certain technological hurdles. The first is that the geopolymer slurry should have an appropriate viscosity. If the slurry is too fluid, the coating on the template struts might be too thin or discontinuous. If the slurry is too viscous, the slurry may not thoroughly permeate the template. Secondly, the drying should be regulated to minimise cracking due to capillary strains and shrinkage. Third, the polymeric template has to be removed without collapse of the geopolymer skeleton. Finally, the mechanical strength is limited by the high proportion of open pores in the final structure, which is strictly constrained compared to denser syntactic foams or mildly foamed materials. These constraints suggest that replica processing is not the best option for high strength building blocks but is very significant where permeability and open cell design are more essential than load bearing capacity.

The replica pathway is unique in comparison to other pore producing techniques. The open-cell network is enforced by the template and is thus significantly more dependable in terms of pore interconnectivity than the direct and microwave-assisted foaming. It also allows bigger and more continuous flow channels than reactive emulsion templating, which often results in smaller droplet-derived pores and hierarchical mesoporosity. However, replica processing has less latitude for designing graded, anisotropic or application specific geometries than additive manufacturing. Hence its usefulness is in constructing strong, highly permeable open cell structures from very basic templates, rather than in fully programmable design.

The replica approach is most suitable for porous geopolymer derived materials where open porosity and fluid transport control the performance requirements. Its key benefits are excellent interconnectivity of pores, template-controlled macroporosity and appropriateness for filtration or catalyst-support applications.^145,146^ Its key constraints are its dependency on templates, the requirement of extra processing steps, removal of polymer, shrinkage, energy consumption during sintering, and relatively poor compressive strength at extremely high porosity.^145,146^ Hence the replica approach should not be seen as a broad way of obtaining cheap thermal insulation or lightweight structural parts, but rather as a directed route to effective open-cell materials.

#### 3.2.4. Sacrificial filler method

The sacrificial filler technique is a template removal method in which a sacrificial phase is added to the geopolymer matrix and then removed to create porosity. In contrast to direct foaming where holes are formed by gas bubbles during setting, a solid or frozen pore producing phase is introduced in sacrificial filler processing. The size, shape and spatial distribution of this phase may be chosen prior to hardening. This sacrificial phase is removed after curing or consolidation of the geopolymer matrix by dissolution, melting, sublimation, thermal breakdown or chemical extraction, leaving holes that replicate the original geometry of the detachable phase. Thus, the approach offers a greater level of control over pore size and form than gas-based foaming, but is less digitally programmable than additive manufacturing.

This approach is a middle ground between the replica method and additive manufacturing. It is similar to the replica route, in that it involves removing a temporary template. But the sacrificial filler approach can be employed with scattered particles, fibres, ice crystals or purposely created polymeric templates, whereas replica processing normally reproduces a continuous open-cell sponge. It can therefore produce isolated pores, partially linked porosity or fully interconnected channels depending on filler loading, particle packing, percolation and template shape. The major strength of the technique is its flexibility. The key disadvantage is that porosity is only achieved after an additional removal stage which may cause shrinkage, breaking, incomplete extraction, contamination or higher processing cost.

Freeze-casting (also known as ice templating) is an essential category of sacrificial filler processing. In this pathway, the water in the geopolymer solution is frozen, and the developing ice crystals serve as temporary templates. After freezing, the ice is removed, usually by sublimation, leaving the geopolymer structure with the shape of the ice crystals. This method was employed by Papa *et al.* to develop orientated and hierarchical porosity in geopolymer derived materials.^147,148^ The importance of this pathway resides in its capacity to generate anisotropic and lamellar porosity, which are challenging to achieve by direct foaming. Unlike round or irregular gas generated holes, ice templating may also generate aligned channels, whose orientation is dictated by the freezing direction and heat gradient.^147,148^ This renders freeze-casting viable for applications requiring directed transport, capillary flow or anisotropic mechanical and thermal characteristics.

However, freeze casting also exemplifies the general shortcomings of sacrificial filler approaches. The pore design is quite sensitive to freezing rate, temperature gradient, solid loading and suspension stability. Finer pores may be produced by rapid freezing whereas wider lamellar channels may be favoured by longer freezing, both of which are very system-dependent. In addition, tensions due to drying or sublimation tend to produce damage to the fragile green body. Ice templating offers more control over pore orientation than direct foaming, but needs careful management of the heat processing and is not as easy to scale up for big structural parts.

Franchin *et al.* demonstrated a more deterministic version of the sacrificial filler idea by using 3D-printed polylactic acid (PLA) templates to generate customised 3D pore networks in geopolymer-based materials.^209^ Once the geopolymer matrix was built around the printed PLA structure, the polymer template was dissolved to provide a linked macroporous network.^209^ This is crucial since it blends sacrificial templating with digital design: the pore architecture is not determined by the particle size or spontaneous phase morphology anymore but by the geometry of the printed template. This allows for a considerably better control on pore connection and spatial organization than direct foaming. It provides more complicated and bespoke pore geometries in comparison to standard replica processing and commercially available sponge templates.

When the particles are removed as distinct entities, the remaining pores will tend to be substantially isolated until the volume fraction of filler is beyond a percolation threshold. Structures of this kind could be effective for lowering density or thermal conductivity, but may not have sufficient connection for filtering or catalysis. The approach may create continuous channels and linked macroporosity using fibres, ice crystals or 3D-printed templates, making it more appropriate for transport-driven applications. Thus, the functional performance of geopolymers made from sacrificial fillers is less dependent on overall porosity and more dependent on the sacrificial phase creating a linked pore network before its removal. This fits with the general idea that open, interconnected holes are necessary for fluid movement, filtration, catalysis and adsorption, but closed or isolated pores are preferable for thermal insulation.

The approach also has practical limits that need to be taken into account when assessing scalability. The sacrificial phase may need to be removed by water washing, solvent extraction, freeze-drying, thermal breakdown or chemical treatment. Each technique has varied consequences for cost, time, energy demand, environmental impacts and dimensional stability. For example, water soluble fillers are easy to remove but may cause an increase of the drying shrinkage or residual ions. Polymeric templates can be used to provide a great control of the shape but may require the use of solvents for the dissolution or thermal burnout. Ice templating does not need any organic templates but it needs freezing equipment and controlled drying. The extra processing stages make sacrificial filler processing less suitable for low-cost bulk insulating products than direct foaming or lightweight filler integration. However, they are justified in the case of high-value porous components when regulated open channels or hierarchical pore structures are desired.

Sacrificial filler processing is more adaptable than the replica approach, but often less scalable than direct foaming *vs.* other production procedures. It is capable of producing pore geometries that are hard to access by gas evolution and approaches the architectural control of additive manufacturing, in conjunction with 3D-printed templates.^209^ However, unlike direct ink writing, the final item is not built up totally in a layer-by-layer fashion from a geopolymer ink, but instead the pore network is specified by a detachable phase contained in the matrix. This may diminish the rheological requirements for printing the geopolymer itself although it shifts the complexity to the manufacturing and removal of templates. Therefore, the process is especially helpful when the desired pore architecture is too intricate to achieve *via* direct foaming, but when complete geopolymer additive manufacturing is not needed or is unfeasible.

Roviello *et al.* also demonstrated a less conventional sacrificial-templating route based on dispersed epoxy-resin domains in a metakaolin geopolymer matrix.^210^ Up to 25 wt% resin was incorporated without marked macroscopic segregation, whereas higher contents approached the compatibility limit. Thermal treatment at 800 °C removed the organic phase and generated a disordered hierarchical pore system extending from nanometre-scale matrix porosity to micrometre-sized cavities corresponding to the former resin domains; the cumulative pore volume increased by approximately fivefold relative to the untreated hybrid. The study demonstrates that chemically compatible organic domains can function as removable pore-forming phases and generate multiscale porosity.^210^ Nevertheless, pore geometry remained irregular rather than deterministically replicated, and the severe burnout treatment increased energy demand and induced crystallisation of the inorganic matrix. Therefore, this route is more appropriate for producing hierarchical porous ceramic-like bodies than for applications requiring preservation of the original geopolymer structure or precise macropore architecture.

In general, the sacrificial filler approach is a versatile way to produce porous geopolymer materials with adjustable pore size, shape and connectivity. The primary benefits are the extensive range of the removable phases, the possibility to manufacture non-spherical and interconnected porosity and the compatibility with hybrid techniques like as freeze-casting or 3D-printed sacrificial templates.^203–205^ The primary drawbacks include the additional stage of removal, probable shrinkage or cracking, partial extraction of the template, more processing time and a possible environmental load. Therefore, sacrificial filler processing is best suited for functional porous components, catalyst supports, filters and architected monoliths when pore shape and connection warrant the additional processing complexity. Simpler approaches such as direct foaming or lightweight filler inclusion are often more practicable for low-cost lightweight building blocks or bulk insulating materials.

## 4. Comparative analysis of porous geopolymer fabrication methods

From the previous sections it can be shown that porous geopolymer materials cannot be compared only on the basis of the total porosity. Geopolymers with identical porosity can have substantially variable mechanical strength, thermal conductivity, permeability and durability depending on how the pores are created (open or closed) and the distribution of the holes in the geopolymer matrix. In this paradigm, the processing route regulates the pore-forming mechanism, the mechanism dictates the pore size, shape, connectivity and spatial distribution, and the resulting pore architecture defines the application-specific performance.

The techniques for manufacturing addressed in this paper can be structured along a continuum from stochastic pore production to deterministic architectural design. Dynamic pore generation by gas or vapour expansion in a setting geopolymer matrix is achieved *via* direct foaming and microwave aided foaming. By lightweight filler is meant the addition of already existing hollow or porous particles to the matrix. Reactive emulsion templating, replica processing and sacrificial filler procedures utilise removable stages to determine pore architecture. Digital prescription of pore geometry affords the greatest degree of design control. This categorisation is beneficial as it relates the practical choice of fabrication process to the desired material function, rather than treating all porous geopolymers as a single material class.

### 4.1. Comparison of representative systems

A direct comparison of published data needs to be done very carefully as various precursors, activators, curing regimens, sample sizes and testing procedures are used in the investigations. Nevertheless, quantifiable numbers are needed to determine the true performance range of any manufacturing technique. Table 1 summarises example systems described in this research and compares the available data on porosity, density, compressive strength, thermal conductivity and surface area.

**Table 1:** Representative performance ranges reported for porous geopolymer systems produced by different fabrication routes

Fabrication route	System	Porosity/pore features	Density	Compressive strength	Thermal conductivity/surface area	Critical comment	Reference
Direct foaming	Fly ash + sodium silicate + H_2_O_2_	79.9–81.2%	0.24 g cm^−3^ for highly porous sample	0.67–0.82 MPa	0.0744 W m^−1^ K^−1^	Very low thermal conductivity was achieved, but strength remained below 1 MPa; system is more suitable for insulation than load-bearing use	Feng *et al.*^99^
	High-magnesium nickel slag + fly ash + H_3_PO_4_ + H_2_O_2_/Al	H_2_O_2_ produced larger pore volume; Al produced finer pores	H_2_O_2_: 0.64–0.82 g cm^−3^; Al: 0.73–1.13 g cm^−3^	Up to 3.6 MPa after 7 days; higher late-age strength was generally obtained with Al powder	H_2_O_2_: 0.25 W m^−1^ K^−1^; Al: 0.19 W m^−1^ K^−1^	Aluminium gave a better insulation-strength balance because finer pores reduced thermal conductivity while preserving higher strength	Yang *et al.*^157^
	PWS + metakaolin + H_3_PO_4_	29.4%; relatively uniform macropores; porosity increased with PWS content	—	13.8 MPa	0.076–0.093 W m^−1^ K^−1^	Strong improvement in thermal insulation, but strength decreased as foaming increased, clear strength-insulation trade-off	Majdoube *et al.*^158^
Reactive emulsion templating	Alkaline geopolymer + triglyceride oil templating	Hierarchical meso-/macroporous structure	—	—	BET surface area 55–124 m^2^ g^−1^	High surface area functional materials than to construction insulation	Medpelli *et al.*^162^
	Geopolymer/zeolite composite	Interconnected macropores of several tens of µm	0.55 g cm^−3^	—	BET surface area 120 m^2^ g^−1^	Study focused on surface area and pore hierarchy, not on mechanical/thermal performance	Gossard *et al.*^165^
Microwave-assisted foaming	Fly ash geopolymer + 200–850 W	Porous structure formed after 1 min at 200–850 W; 5.77 cm^3^ open pores volume	0.8–1.6 g cm^−3^	—	55–126 m^2^ g^−1^	Study focused on surface area and pore hierarchy, not on mechanical/thermal performance	Onutai *et al.*^172^
	Self-ignition coal gangue alkali-activated foam + 750–800 W	Total porosity 74–84%	0.47 g cm^−3^	1.89–2.04 MPa	0.104–0.155 W m^−1^ K^−1^	Authors compared direct foaming with microwave-assisted foaming; microwave foaming process is significantly shorter than direct foaming method	Li *et al.*^174^
	Fly ash + NaHCO_3_ + NaOH + Na_2_SiO_3_	Total porosity ∼60%	0.35–0.95 g cm^−3^	1.60 MPa for microwave foamed samples	—	Microwave foaming gives high porosity rapidly	Zheng *et al.*^175^
Lightweight filler incorporation	Fly ash cenospheres	Filler derived closed porosity	0.69 g cm^−3^	∼5 MPa	—	Study showed a much better strength-density balance than highly gas-foamed systems	Peng *et al.*^192^
Additive manufacturing	Printer geopolymer lattices + DIW	Designed lattice porosity; 1.2 mm – 46.59%	—	1.2 mm – 11.5 MPa	—	AM should be compared by architectural control and load path design, not only by total porosity	Franchin *et al.*^205^
		1.6 mm – 59.16%		1.6 mm – 4.7 MPa			
		2.4 mm – 71.73%		2.4 mm – 2 MPa			
	Powder-bed, sodium-based geopolymer	Apparent porosity ∼57 vol%	—	0.7–0.9 MPa; up to 10 MPa after alkaline post-processing	—	Powder-based 3D printing gives uniform porosity but low initial strength; post-processing is needed	Xia and Sanjayan^206^
	Extrusion AM; geopolymer/GO composites	∼50% apparent porosity	—	Up to 110 MPa	0.02–0.05 W m^−1^ K^−1^	Study focuses mainly on compression strength	Zhong *et al.*^196^
Replica method	PU sponge template	Interconnected cellular structure with 87.96 vol%	—	∼0.85 MPa	—	Excellent open porosity but low strength because load is carried by thin struts	Kovářík *et al.*^145^
	Cordierite geopolymer/PU foam	Total porosity ∼89% after sintering	—	0.47 MPa after sintering	—	Post-treatment improved strength while retaining high open porosity, but strength remained below 1 MPa	Bai *et al.*^146^
Sacrificial filler method	Ice templating	Total porosity 53–83 vol%	—	7 MPa for samples with 83% porosity	—	Unlike many highly porous foams, ice-templated samples reportedly retained relatively high compressive strength	Papa *et al.*^147,148^
	3D printed PLA template	Total porosity 66–71 vol%; interconnected mm-scale pores	—	∼8.5 MPa	—	Strong example of designed interconnected porosity with better strength	Franchin *et al.*^209^

It should be emphasised that the parameters reported in Table 1 are not always directly equivalent. Total porosity, open porosity, image-derived pore area fraction, mercury-intrusion pore volume and BET specific surface area describe different aspects and length scales of the pore system. Likewise, compressive strength values are influenced by precursor chemistry, activator composition, curing regime, specimen geometry, loading rate and test age. Therefore, the values compiled in Table 1 should be interpreted as representative performance ranges rather than as a strict ranking of fabrication methods. Comparisons are most meaningful when systems of similar density, curing history and testing conditions are considered. Where available, the strength to density balance, thermal conductivity within comparable density ranges and method-specific pore descriptors are discussed instead of relying on porosity alone.

As seen in Table 1, high porosity alone is not a good predictor of quality of material. The direct foaming with H_2_O_2_ can yield low-density geopolymers with poor thermal conductivity, but, when the cellular structure is greatly extended or heterogeneous, the compressive strength is generally lower than 1 MPa.^99^ This is not a failure of the procedure, but rather determines the practical window of applicability. These materials are ideal for applications where the primary objective is insulation, but cannot be directly compared to denser lightweight composites or printed constructions designed to maintain mechanical strength.

A similar trade-off between strength and insulation is seen in phosphate-based foams. Foaming based on aluminium *vs.* H_2_O_2_ showed that the aluminium based one led to smaller pores and a better balance between heat conductivity and strength whereas H_2_O_2_ delivered a higher density decrease but less advantageous mechanical performance.^157^ The thermal conductivity of phosphate washing sludge was also much reduced, although this improvement was coupled with a loss in compressive strength due to the increase of porosity.^158^ These examples illustrate that the choice of foaming agent should not be based just on gas yield, but rather on the effects of gas evolution on pore size, matrix continuity and strength retention.

The addition of lightweight filler creates a new balance of performance. Peng *et al.* described a fly ash cenosphere system with a density of 0.69 g cm^−3^ and a compressive strength of around 5 MPa.^191^ This is substantially more than the strength of many fully expanded gas foamed systems. This is a structural reason: the pores are trapped in hollow particles, not as the result of coalescing gas bubbles.

Therefore, the geopolymer foams based on fillers are more suitable for light building items when moderate strength is still required.

The chart also indicates that compressive strength or thermal conductivity should not be the key judging parameters. The key benefit is a large surface area and hierarchical pore structure. Specific surface areas of 55–124 m^2^ g^−1^ were reported by Medpelli *et al.*^162^ and geopolymer/zeolite monoliths with a BET surface area of about 120 m^2^ g^−1^ and a mesopore volume of about 0.55 cm^3^ g^−1^ were developed by Gossard *et al.*^165^ These values bring them closer to adsorption, catalysis and fixed-bed applications than standard building insulation.

Microwave aided foaming can best be regarded as a quick processing approach. Some reported systems can approach high porosity, usually 74–84 vol% with limited thermal conductivity in some compositions.^174,175^ The pore shape, however, is frequently less regular than in template generated systems. Microwave foaming is potential for quick manufacturing of lightweight aggregates or insulation components, while greater control of moisture distribution, sample size and heating uniformity is required before it can be considered as a completely scalable approach for big building elements.

Why pore architecture is as crucial as porosity is shown by additive manufacturing and sacrificial-template approaches. Powder-based printed geopolymers can exhibit high apparent porosity but poor initial strength, while extrusion-based geopolymer composites can achieve substantially greater compressive strength when the matrix is thick and the printed structure is controlled.^196,206^ Besides, the ordered pore network and continuous load-bearing skeleton allow freeze-cast and PLA-template-derived geopolymers to retain relatively high strength at high porosity.^145,146,209^ These examples illustrate that planned porosity may give higher mechanical efficiency than random produced porosity.

### 4.2. Main trade-offs between fabrication routes

Table 1 shows that the data provide three significant trade-offs. The first is a trade-off between decreasing density and strength. Both direct and microwave foaming can result in high porosity and low heat conductivity, but the mechanical performance of the foams is greatly influenced by the fine and uniform or broad and uneven pore distribution. Rapid creation of gas or vapour can be efficient in reducing density, but if matrix hardening is not coordinated with pore development, the outcome is a fragile cellular structure.

The other trade-off is between pore connection and strength. Open interconnecting pores are useful for filtration, catalysis and adsorption but lower the effective load-bearing cross-section. This explains why replica generated foams can exhibit very high open porosity but very poor compressive strength. They are not valuable for structural load-bearing capability but for their permeability and available pore networks. RET follows a similar rationale, except its benefit is more directly tied to the hierarchical porosity and surface area.

The third trade-off is between architectural control and complexity of processing. Direct foaming and filler integration are less complicated and more compatible with normal building operations. However, they provide poor control over the pore shape compared to template-based or digitally developed approaches. Replication, sacrificial filler and AM methods can lead to more regulated pore networks but involve additional processes such as template manufacturing, impregnation, extraction, sintering, solvent treatment, rheology control or layer-by-layer printing. Thus, the improved control over the pores often results in increased cost, longer processing time or more difficult scale-up.

These trade-offs explain why no single fabrication method can be considered universally superior. Direct foaming is suitable for low-cost insulation, because it is easy and scalable. Lightweight fillers are promise for lightweight building composites because of enhanced strength-density balance. RET, replica and sacrificial filler technologies are better appropriate for functional porous materials requiring open porosity, surface area or permeability. AM is the most versatile technique for planned structures, but is currently more appropriate for specialist components than mass manufacturing.

### 4.3. Application-based method selection and remaining challenges

The desired pore structure for thermal insulation is often a closed or semi-closed pore structure, characterised by low density and minimal moisture absorption. Therefore, the most important ways are direct foaming, microwave foaming, and the introduction of lightweight fillers. However, direct foaming has to be carefully controlled with respect to surfactants, viscosity and setting time to avoid major connected faults. The water quantity and consistency of heating also need to be controlled in microwave foaming. Filler-based systems are more predictable but their effectiveness is dependent on filler availability, interfacial bonding and resistance to crushing during mixing.

In filtration, catalysis and adsorption, minimum density is less significant than open and interconnected porosity. RET, replica processing, sacrificial filler techniques and AM are more suitable for these applications as they can produce accessible pore networks. The downside is that these pathways are typically more difficult and less desirable for low-cost mass building items. Their usage is more warranted when the porous geopolymer is intended as a functional monolith, as catalyst support, as adsorbent or as a permeable ceramic-like component.

The most promising techniques for lightweight structural or semi-structural building materials are those that reduce density without disrupting matrix continuity. In this regard, lightweight filler and properly stabilised direct foaming are more realistic than excessively open replica structures. AM might be useful for tailored load-bearing or multifunctional components, but broader deployment requires printable geopolymer mixes with stable rheology, reliable interlayer bonding and controlled curing.

The next key difficulty is to transition from reporting stand-alone material compositions to application-specific design guidelines. Future research should report porosity in addition to pore size distribution, open/closed pore ratio, density, compressive strength, thermal conductivity and where appropriate permeability or BET surface area. Without this whole data set it is difficult to meaningfully compare techniques. The next step of porous geopolymer research should thus be less concerned with the production of “high porosity” as a generic aim, but instead focus on matching pore design to desired function.

To clarify the application window of each route, Table 2 summarises the dominant pore architecture, approximate reported compressive strength and most suitable application areas for the main porous geopolymer fabrication methods.

**Table 2:** Application-oriented selection of porous geopolymer fabrication method

Fabrication method	Dominant pore architecture	Compressive strength	Most suitable application	References
Direct foaming with H_2_O_2_	Mostly closed or mixed pores; without surfactants become irregular and interconnected is gas release is too rapid	Usually <1–3 MPa for highly porous/low density foams	Thermal insulation, ultra-lightweight blocks, non-load bearing panels, fire-resistant lightweight components	50, 93–100, 110, 114, 115, 123–125, and 144
Direct foaming with metallic powders	Mixed pores	∼1–5 MPa, depending on activator chemistry and metal dosage	Thermal insulation, ultra-lightweight blocks, non-load bearing panels, fire-resistant lightweight components	126, 127, and 149–157
Direct foaming with alternative gas-releasing agents	Mixed pores; more controlled gas release	∼1–7 MPa	Thermal insulation, ultra-lightweight blocks, non-load bearing panels, fire-resistant lightweight components	139–143 and 158
Reactive emulsion templating	Mainly open and hierarchical porosity	—	Adsorption, catalysis, filtration, fixed-bed monoliths	160–165
Microwave-assisted foaming	Irregular mixed pores	∼1–2 MPa	Thermal insulation, ultra-lightweight blocks, non-load bearing panels, fire-resistant lightweight components	166–175
Embedding lightweight fillers	Mostly closed or filler derived pores	∼5 MPa	Thermal insulation, ultra-lightweight blocks, non-load bearing panels, fire-resistant lightweight components	176–192
Additive manufacturing	Designed open, closed or lattice porosity	0.7–110 MPa	Custom filters, architected lattices, graded porous components, acoustic panels	193–208 and 211
Replica method	Highly open and interconnected macropores	Usually, <1 MPa	Filters, membranes, catalyst supports	144–146
Sacrificial filler method	Isolated, open or interconnected pores	∼7–8.5 MPa	Filters, catalyst supports, adsorbents, permeable monoliths and functional components requiring controlled open or interconnected porosity	147, 148, 209 and 210

To facilitate the selection of an appropriate fabrication route, the main methods were compared using five criteria: pore-architecture control, pore interconnectivity, processing simplicity, scalability and mechanical performance potential. Each criterion was assigned a semi-quantitative score from 1 (very low) to 5 (very high) based on the critical literature analysis presented in Sections 3 and 4 and the data summarised in Tables 1 and 2. The resulting decision matrix is presented in Fig. 12, and represents the authors' subjective expert assessment, based on a critical analysis of the literature published in this field.

**Fig. 12:**
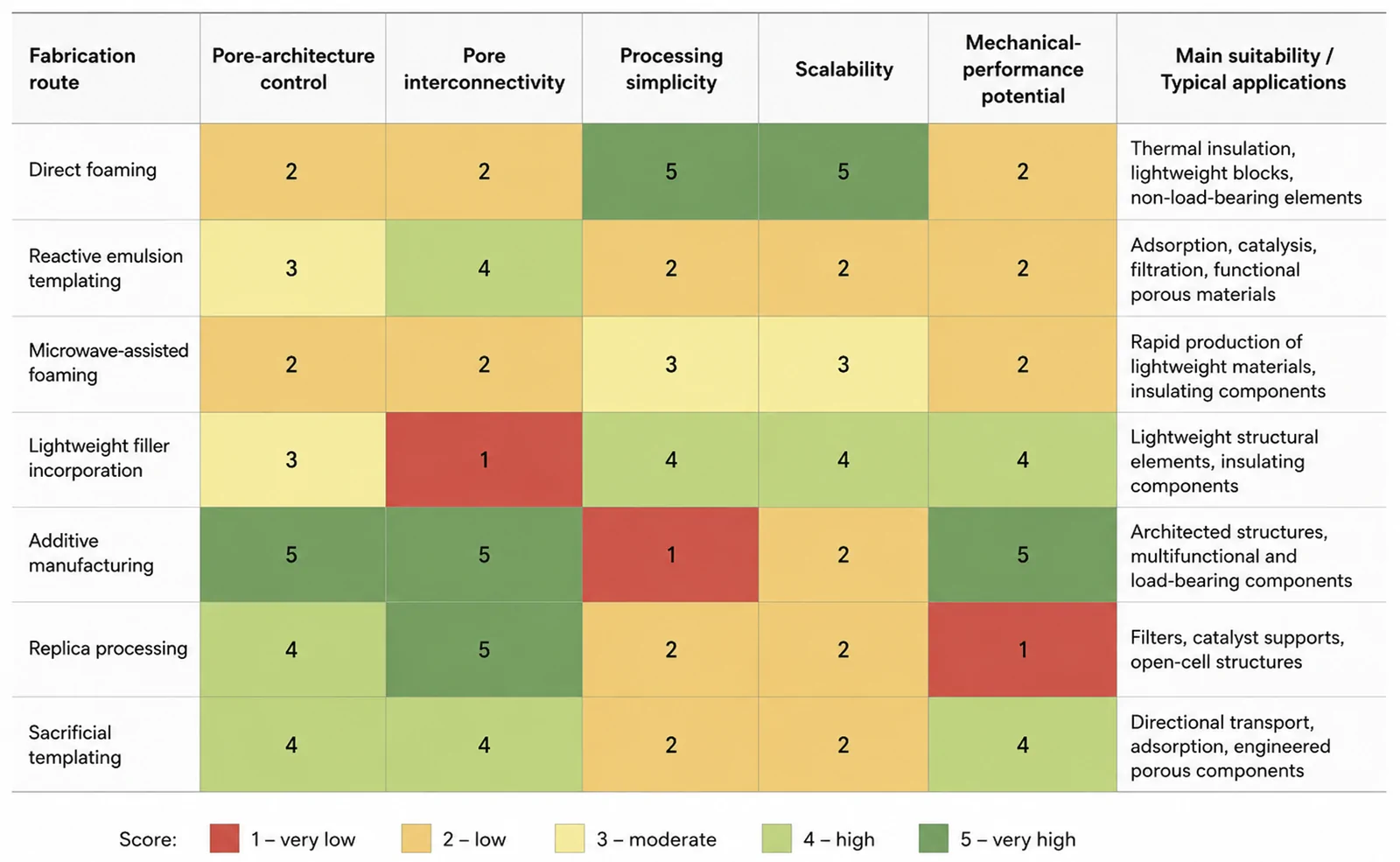
Semi-quantitative decision matrix comparing the principal fabrication routes for porous geopolymer-based materials in terms of pore-architecture control, pore interconnectivity, processing simplicity, scalability and mechanical performance potential. Scores range from 1 (very low) to 5 (very high) and represent general method-level tendencies derived from the critical literature synthesis rather than statistical averages of directly comparable experimental results.

Fig. 12 demonstrates that no single fabrication route is superior in all categories. Direct foaming offers the highest processing simplicity and scalability but provides limited pore-architecture control and mechanical performance. Additive manufacturing enables the greatest control over pore geometry and offers high mechanical performance, although its processing complexity and limited scalability remain important drawbacks. Replica processing and reactive emulsion templating are particularly suitable for producing highly interconnected pore networks, whereas lightweight filler incorporation provides a favourable balance between scalability and mechanical performance. Sacrificial templating occupies an intermediate position, combining relatively high architectural control and mechanical potential with additional template-removal requirements. Therefore, the selection of a fabrication route should be based on the intended application and the required balance between structural control, pore accessibility, mechanical performance and production feasibility.

Overall, the analysed results indicate that the production of porous geopolymer should be seen as a method-selection challenge rather than a straightforward foaming problem. The pore architecture is controlled by the processing method, the balance between strength, insulation, permeability and surface accessibility is controlled by the pore architecture and the application for which the material is designed controls which balance is acceptable. This is the main rationale for the selection among direct foaming, filler integration, RET, microwave foaming, replica processing, sacrificial templates and additive manufacturing.

## 5. Conclusion

This review has examined the main strategies for introducing controlled porosity into geopolymer matrices, linking the chemistry of geopolymerisation to the engineering of pore architecture.

The choice of aluminosilicate precursor and activator governs the gel chemistry, which in turn affects the compatibility with different foaming routes. Among the fabrication methods, direct foaming remains the simplest and most cost-effective way to achieve high porosity and low density, although it often requires surfactants to stabilise the wet foam and avoid an overly heterogeneous pore structure. For applications that demand precisely controlled, interconnected pores, such as filtration or catalysis, templating methods, such as replica, sacrificial filler, emulsion templating, offer superior architectural control. Additive manufacturing, especially direct ink writing, provides unmatched freedom to design non-stochastic pore networks, but the rheological demands of geopolymer inks still pose challenges. Microwave foaming stands out a rapid, energy-efficient alternative that couples foaming and curing in minutes.

Overall, the choice of fabrication method must be driven by the intended application; thermal insulation favours closed-pore structures from direct foaming or syntactic foams whereas fluid-transport applications require open, well-connected pores best achieved by replication or emulsion templating. Future progress will likely come from hybrid approaches, the development of greener foaming agents, and a deeper understanding of the rheological and kinetic control needed to scale these technologies from laboratory to industrial production.

## Conflicts of interest

There are no conflicts to declare.

## Data Availability

No primary research results, software or code have been included and no new data were generated or analysed as part of this review. All information discussed in the article was obtained from the published literature cited in the reference list.
